# Implementation of DHIS2 for Disease Surveillance in Guinea: 2015–2020

**DOI:** 10.3389/fpubh.2021.761196

**Published:** 2022-01-20

**Authors:** Eileen Reynolds, Lise D. Martel, Mamadou Oury Bah, Marlyatou Bah, Mariama Boubacar Bah, Barry Boubacar, Nouhan Camara, Yero Boye Camara, Salomon Corvil, Boubacar Ibrahima Diallo, Ibrahima Telly Diallo, Mamadou Kadiatou Diallo, Mamadou Tafsir Diallo, Telly Diallo, Siba Guilavogui, Jennifer J. Hemingway-Foday, Fatoumata Hann, Abdoulaye Kaba, Almamy Karamokoba Kaba, Mohamed Kande, Diallo Mamadou Lamarana, Kathy Middleton, N'valy Sidibe, Ousmane Souare, Claire J. Standley, Kristen B. Stolka, Samuel Tchwenko, Mary Claire Worrell, Pia D. M. MacDonald

**Affiliations:** ^1^Research Triangle Institute International, Durham, NC, United States; ^2^Division of Global Health Protection, Centers for Disease Control and Prevention, Atlanta, GA, United States; ^3^Research Triangle Institute International, Conakry, Guinea; ^4^Ministry of Public Health and Hygiene, Conakry, Guinea; ^5^African Field Epidemiology Network, Conakry, Guinea; ^6^Center for Global Health Science and Security, Georgetown University, Washington, DC, United States; ^7^Department of Epidemiology, Gillings School of Global Public Health, University of North Carolina, Chapel Hill, NC, United States

**Keywords:** epidemic-prone diseases, disease notification, epidemiological monitoring, public health informatics (MeSH), Guinea (Conakry), health informatics and information systems, developing and transition countries

## Abstract

A robust epidemic-prone disease surveillance system is a critical component of public health infrastructure and supports compliance with the International Health Regulations (IHR). One digital health platform that has been implemented in numerous low- and middle-income countries is the District Health Information System Version 2 (DHIS2). In 2015, in the wake of the Ebola epidemic, the Ministry of Health in Guinea established a strategic plan to strengthen its surveillance system, including adoption of DHIS2 as a health information system that could also capture surveillance data. In 2017, the DHIS2 platform for disease surveillance was piloted in two regions, with the aim of ensuring the timely availability of quality surveillance data for better prevention, detection, and response to epidemic-prone diseases. The success of the pilot prompted the national roll-out of DHIS2 for weekly aggregate disease surveillance starting in January 2018. In 2019, the country started to also use the DHIS2 Tracker to capture individual cases of epidemic-prone diseases. As of February 2020, for aggregate data, the national average timeliness of reporting was 72.2%, and average completeness 98.5%; however, the proportion of individual case reports filed was overall low and varied widely between diseases. While substantial progress has been made in implementation of DHIS2 in Guinea for use in surveillance of epidemic-prone diseases, much remains to be done to ensure long-term sustainability of the system. This paper describes the implementation and outcomes of DHIS2 as a digital health platform for disease surveillance in Guinea between 2015 and early 2020, highlighting lessons learned and recommendations related to the processes of planning and adoption, pilot testing in two regions, and scale up to national level.

## Introduction

The West Africa Ebola outbreak of 2014–2016 highlighted the need for public health systems strengthening across the region, including enhancing capacities to routinely monitor epidemic-prone disease trends and effectively and efficiently detect outbreaks (1, 2). During the Ebola outbreak, the use of multiple independent surveillance digital health platforms, and the resulting lack of integrated data collection, timely aggregation, and sharing of data, hampered the response in affected countries (3–5). In Guinea, where the outbreak started, the health system had deteriorated over a period of 15 years due to political instability including civil unrest and regional armed conflicts, as well as fragmented approaches to health assistance from global partners and initiatives (6). Guinea's Ministry of Health (MOH) cited weak capacity in data management as one of several factors contributing to the delayed detection and response to the Ebola outbreak in 2014 (7). A 2014 study specifically outlined weaknesses in Guinea's overall health information system (8).

A robust epidemic-prone disease surveillance system is a core component of public health infrastructure and supports compliance with the International Health Regulations (IHR) (9). Lessons learned across countries indicate that use of appropriate technologies such as digital health platforms that use open-source software and online systems to track surveillance information encourages quicker, more accurate reporting and use of information (10). Digital health platforms that are coordinated, interoperable, and standardized provide a foundation for robust disease surveillance and reporting (9). One of these, the District Health Information System Version 2 (DHIS2), an open source configurable platform (11), has been implemented in over 73 low and middle income countries for routine health information reporting (12). Several countries are also using DHIS2 for epidemic-prone disease surveillance (13) as the system enables integrated reporting and analysis of aggregate and case-based surveillance data across diseases. Examples of countries in Africa using DHIS2 for epidemic-prone disease surveillance include Sierra Leone (14), Mali (15), Rwanda (16), Uganda (17), and Tanzania (18).

As a routine health information system, DHIS2 gathers data at regular intervals (usually no greater than annually) from public and private health facilities to document health status, services provided, and resources available (19). As a disease surveillance system, DHIS2 provides the opportunity for early warning and monitoring of disease outbreaks and has the ability to collect and disseminate data immediately as cases are identified. DHIS2 can easily be adapted to meet the changing requirements of existing tools such as the World Health Organization's (WHO) Africa Region's Integrated Disease Surveillance and Response (IDSR) framework (20), and the International Health Regulations (IHR). In addition, the DHIS2 platform has several features that facilitate interoperability. These include the international standard for aggregate data exchange, ADX.^1^

**Figure 1:**
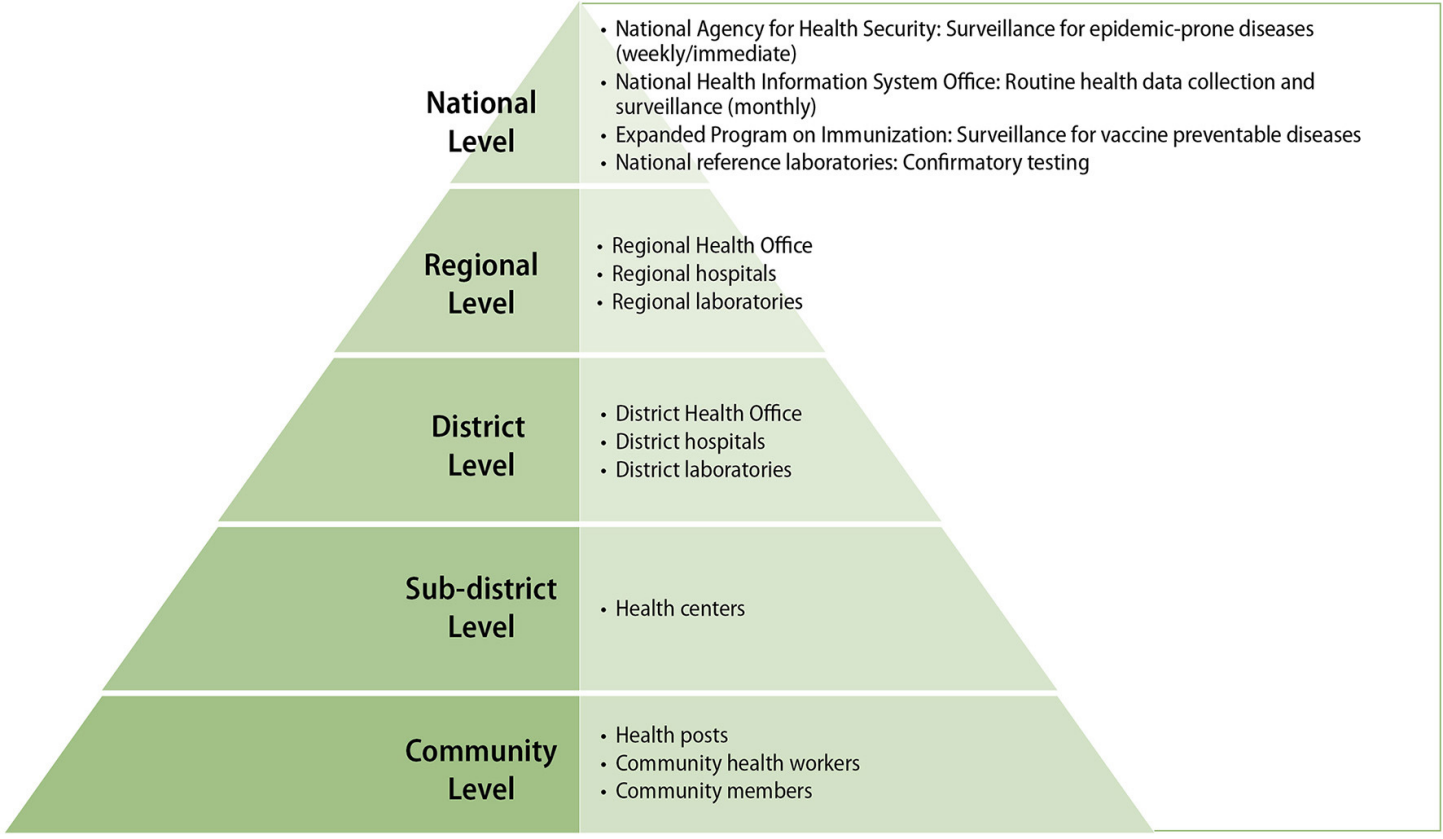
Guinea's health pyramid structure.

In 2015, to better prepare for future outbreaks and following recommendations of the 2014 study, Guinea's MOH developed a detailed plan to strengthen epidemic-prone disease surveillance (7). The plan was based on a health “pyramid” structure composed of community-level health posts and centers reporting to district and intermediate (regional) health offices, which in turn report to appropriate national level health authorities (see Figure 1 Guinea's Health Pyramid Structure) (7).

The plan recommended the review and updating of Guinea's national technical guidelines for IDSR, which contains the country's list of priority diseases, and locally-adapted case definitions and reporting guidance (21). In addition, the Ministry of Health developed an overall plan to rehabilitate the health sector considering the impact of the Ebola outbreak, including the recommendation to use DHIS2 to manage data on all epidemic-prone diseases (6). In 2016, the MOH adopted DHIS2 as its national health information systems platform for routine monthly health information reporting. In this paper, we describe the process and lessons learned from the planning, piloting, and scaling up of DHIS2 specifically for epidemic-prone disease surveillance in Guinea from 2015 to 2020.

## Planning and Adoption (2015–2019)

The implementation of DHIS2 for disease surveillance including planning, configuration, piloting, and scale up to all districts took almost 4 years. The initial planning phase, starting in 2015, incorporated: extensive efforts to ensure buy-in first from the Ministry of Health, and later from the Ministry of Livestock and Ministry of Environment, Water and Forests through an inclusive, transparent, consensus-based decision process that included representatives of all levels of the health pyramid; a country-wide assessment of the existing infrastructure, and proactivity to fill the identified gaps; numerous presentations and training opportunities for future users from the national to the district level; and a configuration of the platform with the participation of both information technology and surveillance specialists.

### Stakeholder Engagement and Infrastructure Assessment

Stakeholders' awareness was raised through meetings including DHIS2 demonstrations and attending a DHIS2 Academy (https://www.dhis2.org/academy) to learn more about the platform and its features. A review of the current surveillance system tools and related documentation was conducted through interviews with MOH staff of all levels of the pyramid and observations of surveillance activities. Each district and region was visited to conduct a nationwide information technology infrastructure assessment. The assessment evaluated the availability of: a working space equipped with needed equipment such as computers and printers; staff able to use the equipment; a reliable power source (i.e., electricity, generators, solar panels); and internet connection at the District and Regional Health Offices and hospitals (see Supplementary Material 1 for full assessment criteria). Subsequently, based on the observation that availability of functional basic computing infrastructure and skills were an urgent gap, the WHO and the National Agency for Health Security of the MOH procured and distributed new computers to all District and Regional Health Offices and hospitals in the country, and their personnel was trained in basic computer skills and IDSR.

In November 2016, 42 stakeholder representatives^2^ participated in a 5-day DHIS2 workshop, that presented the requirements and implementation plan for DHIS2, addressed concerns, and encouraged practical and financial buy-in. Concerns raised during the meeting included: lack of adequate human resources at the facility and district levels and turnover of personnel; lack of sufficient communication about the IDSR guidelines and supervision visits; lack of access to internet and electricity; insufficient maintenance of computer equipment; insufficient means of communication for community health workers, for supervision and for feedback to communities; and competing activities at the Regional and District health office levels. In meetings with the National Agency for Health Security, further concerns were raised about the potential for data loss, based on negative experiences with previous information systems projects, and lacking adequate resources to sustain the DHIS2.

The recommendations from the workshop were used to configure the DHIS2 individual case notification forms and aggregate weekly report form. In February 2017, a final half-day planning workshop was held to review and validate the configuration with the MOH leadership and disease surveillance partners and approve a pilot implementation in two regions. The results of the DHIS2 pilot evaluation were shared with the MOH and disease surveillance partners at the weekly disease surveillance meeting at the National Health Security Agency on January 4, 2018, and approval secured to proceed to national scale-up immediately for weekly aggregate reporting and later for individual case reporting as the forms were to be updated.

A sub-group of surveillance experts^3^ then revised, per IDSR, the individual case reporting forms of Guinea's priority diseases/events: acute diarrheal syndrome, acute flaccid paralysis (polio), adverse events following vaccination^4^, anthrax, brucellosis, icteric fever syndrome (yellow fever), influenza-like illness, maternal deaths, measles, meningitis, neonatal/maternal tetanus, rabies, and viral hemorrhagic fever syndrome^5^. The group also revised the format and content of the weekly surveillance report. Information technology specialists, in close collaboration with surveillance experts, completed the configuration of all forms in DHIS2.

In April 2019, the Minister of Health issued a letter to all MOH directorates declaring DHIS2 to be the official health information platform, and that other systems—including for disease surveillance and reporting—should be discontinued, and data migrated to DHIS2 by June 25, 2019, signifying full adoption of DHIS2 (22). However, as of January 2021, while individual case data for epidemic-prone diseases and priority events is reported in DHIS2, parallel reporting of aggregate data in Excel continued.

## Pilot Implementation (2017)

In 2017, for a period of 6 months, a pilot implementation of DHIS2 for disease surveillance, consisting of aggregate weekly and individual case notifications in DHIS2, was conducted to assess implementation needs and challenges at the District and Regional Health Offices, hospitals, the National Agency for Health Security, laboratories, and the Expanded Program on Immunization (see Table 1) for the list of reportable diseases and events that were included in the DHIS2 during the pilot phase.

**Table 1:** Epidemic-prone reportable diseases and events and type of reporting in Guinea, 2017–2020.

**Name of disease/event**	**Differential diagnosis**	**Type of reporting**		**2017 pilot**	**2018–2020 scale-up**
		**Individual immediate**	**Weekly aggregate**		
Acute diarrheal syndrome	Cholera; shigelloses; rotavirus; collective food poisoning	X	X	X	X
Acute flaccid paralysis (polio)	Wild poliovirus; Vaccine-derived poliovirus	X	X	X	X
Adverse events following vaccination	N/A	X			X
Anthrax	N/A	X	X		X
Brucellosis	N/A	X	X		X
Dog bite	N/A		X		X
Snake bite	N/A		X		X
Icteric fever syndrome	Yellow fever; hepatitis; leptospirosis; Congo Crimean fever; Rift Valley fever; dengue fever	X	X	X	X
Influenza-like illnesses^6^	Seasonal flu; avian flu; swine flu		X	X	X
Malaria	N/A		X	X	X
Maternal deaths	N/A	X	X	X	X
Measles	Rubella	X	X	X	X
Meningitis	N/A	X	X	X	X
Neonatal deaths	N/A		X	X	X
Neonatal/Maternal tetanus	N/A	X	X	X	X
Rabies	N/A	X	X		X
Viral hemorrhagic fever syndrome	Ebola; yellow fever; Marbourg; Lassa fever; Rift Valley fever; dengue fever	X	X	X	X

### Pilot Sites

**Figure 2:**
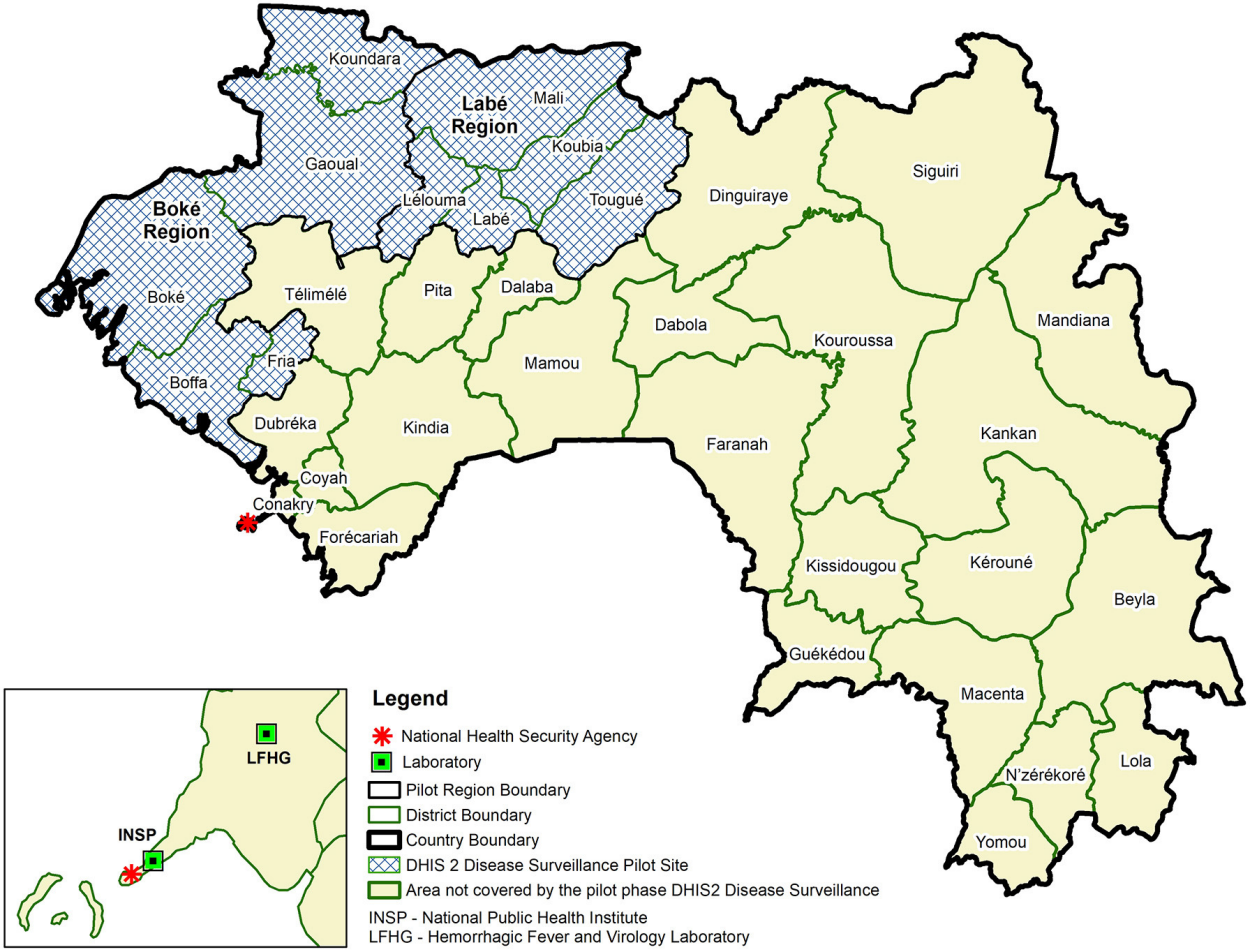
Use of DHIS2 for disease surveillance pilot sites, Guinea, 2017.

The pilot implementation involved two regions and four national level entities (i.e., the National Agency for Health Security, the Expanded Program on Immunization, the National Public Health Institute and Hemorrhagic Fever and Virology Laboratory) (see Figure 2 Use of DHIS2 for Disease Surveillance Pilot Sites, Guinea, 2017). The two regions, Boké and Labé, represent 20%^6^ percent of the population in Guinea (23). Each region has five District Health Offices, one Regional Health Office, and one regional and four district hospitals. To assess the extent to which health facilities could effectively use the system to enter and view their own aggregate weekly data, in addition to these health offices and hospitals, 23 sub-district level health centers were also included in the pilot implementation.

### System Scope and Data Flow

**Figure 3:**
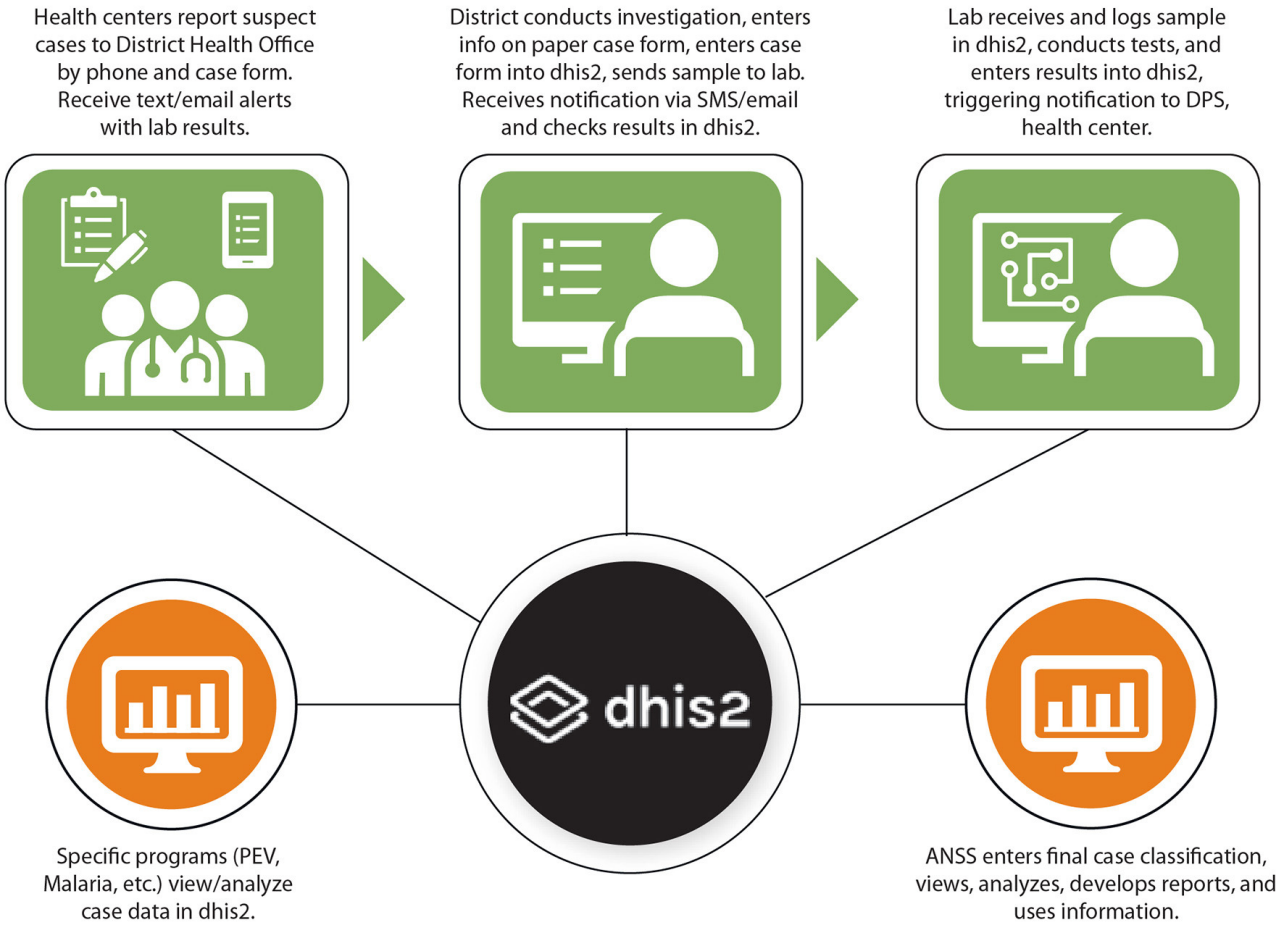
Design of data flow for individual case notifications in DHIS2 tracker in Guinea.

Based on the needs assessments and requirements identified, the pilot disease surveillance system with DHIS2 was designed to enable District Health Offices to enter their aggregate weekly case reports and individual case notifications in DHIS2. A visual representation of how individual case notification information is designed to flow in DHIS2 is provided in Figure 3 Design of Data Flow for Individual Case Notifications in DHIS2 Tracker in Guinea.

### Pilot Trainings and Follow-Up

Twenty-two national-level stakeholders were trained on use of DHIS2 for disease surveillance in April 2017, followed by regional- and district-level stakeholders in Boké and Labé in May 2017. National-level stakeholders trained included representatives from the Office of Strategy and Development and National Health Information System Office of the Ministry of Health, the National Agency for Health Security, the Department for the Prevention and Control of Diseases, the Expanded Program on Immunization, the National Institute of Public Health, and the Hemorrhagic Fever and Virology Laboratory, WHO, CDC, MEASURE Evaluation, International Medical Corps, and the International Organization on Migration. At the regional and district level, two training workshops were held in both regions, focused on different audiences, as summarized in Table 2 DHIS 2 Training in both Boké and Labé by Session, Participant Type, and Topics Covered.

**Table 2:** DHIS 2 training in both Boké and Labé by session, participant type, and topics covered.

**Training session**	**Total trained (both regions)**	**Participants**	**Topics covered**
Session 1	53	Hospital supervisors, laboratory personnel, heads of health centers, heads of centers for treatment of epidemic prone diseases (part of hospital personnel)	• Basic initiation on DHIS2 • Basic computer skills • Data entry and analysis using DHIS2
Session 2	73	Regional Health Office management teams, Prefecture Health Office management teams (Hospital Directors, Heads of Prefecture Health Offices, Chief Medical Officers, Data Managers); Viral Hemorrhagic Fever Laboratory in Conakry, WHO	• Overview of the disease surveillance system and discussion of challenges • Lab sample transportation • DHIS2 weekly disease report data entry • DHIS2 event (maternal deaths) and Tracker (disease case notifications) data entry • Data analysis in DHIS2 • Implementation Roadmap for DHIS2

A refresher training for national reference laboratory staff was provided in August 2017, as the laboratories had not been entering data into the system. In-person supervision visits were conducted in Boké and Labé regions in July, September, and October 2017. During the supervision visits, health workers' skills and understanding in using DHIS2 for disease surveillance were assessed and refresher training was provided where needed. In addition, health workers discussed any challenges and possible solutions. The visits targeted those previously trained on the DHIS2 disease surveillance module and anyone who missed the initial training that should be using the system. Supervision visits generally included five individuals per District Health Office (the Head of the District Health Office, Chief Medical Officer, Statistician, District Hospital Director and District Hospital Statistician) and three people per Regional Health office (the Head of the Regional Health Office, Regional Chief Medical Officer and Regional Statistician). In some cases, laboratory staff, managers of the epidemic-prone disease treatment centers, as well as local WHO and malaria program point persons in the District Health Offices participated. Supervision visits were also conducted to 14 health centers in Boké and five in Labé participating to the pilot and where personnel had been trained on DHIS2.

In addition, regular calls and emails with the District were used as a check-in, to troubleshoot issues, and respond to questions and requests. RTI staff initiated and answered these requests for assistance during the pilot project. There was no formal tracking system for assistance requests.

### Pilot Phase Evaluation

The analysis of the pilot phase includes the following: (1) analysis of weekly aggregate and individual case reporting in the DHIS2; (2) observations from supervision visits; (3) analysis from questionnaires administered to pilot participants (see Supplementary Material 2). To measure whether the system was being used effectively for case reporting, the completeness and timeliness of weekly aggregate reports and the completeness of individual case reporting in DHIS2 were analyzed for both regions. Completeness of individual case reporting in DHIS2 was evaluated by comparing case counts for the same period in the DHIS2 aggregate reports with the number of individual case notifications in DHIS2. Completeness of the weekly disease surveillance reports was measured by the number of health facilities that submitted a report for the week, divided by the total number of facilities expected to report. Timeliness of weekly disease surveillance reports was measured by the number of health facilities that submitted a report by Tuesday of that week, divided by the total number of facilities expected to report. These data were obtained from the national DHIS2.

High rates of completeness and timeliness of aggregate weekly disease reporting indicated sufficient capacity at the district level to use DHIS2 effectively for disease surveillance reporting. The average percentage completeness of weekly aggregate disease reports from health centers that were entered into DHIS2 by participating District Health Offices was 99.1% for the Boké region and 99.9% for the Labé region for epidemiological weeks 22–43, 2017. Over the same period, the timeliness was 80.1 and 85.3% for the Boké and Labé regions, respectively.

However, individual case reports were less complete, as shown in Table 3. Completeness of individual case reporting was highest for acute flaccid paralysis (AFP) (individual case reports represented 75% of aggregate case counts) and lowest for bloody diarrhea/acute diarrheal syndrome (no individual cases were reported, though three were reported in the aggregate reports).

**Table 3:** Comparison of individual case reports as a percentage of weekly aggregate reports across pilot and scale-up periods. Difference in proportions between the pilot and scale up were calculated using Chi-squared tests.

**Disease/event^a^**	**Comparison between individual case reports in DHIS2 and weekly suspect case aggregate reports (%)**		
	**Pilot (May 29-October 29, 2017) (Boké and Labé Regions)**	**Scale up (June 30, 2019-March 7, 2020)** **(All Regions)**	***p-*value**
Acute flaccid paralysis	75% (44/59)	48% (91/189)	*0.003*
Anthrax	Not included in the pilot.	159% (27/17)	N/A
Bloody diarrhea/Cholera/Acute diarrheal syndrome^b^	0% (0/3)	5% (3/55)	0.694
Brucellosis	Not included in the pilot.	0 (0/0)	N/A
Influenza-like illnesses	Not included in the pilot.	22% (105/475)^c^	N/A
Maternal deaths	54% (20/37)	50% (140/281)	0.633
Measles	54% (35/65)	33% (2,147/6,573)	*0.000*
Meningitis	28% (5/18)	51% (214/421)	0.056
Neonatal/maternal tetanus	15% (2/13)	63% (41/65)	***0.002***
Rabies	Not included in the pilot.	3,067% (276/9)	N/A
Viral hemorrhagic fever/viral hemorrhagic fever syndrome^d^	0% (0/0)	113% (9/8)	N/A
Yellow fever/Icteric fever syndrome	34% (18/53)	49% (74/151)	0.059

*Significant differences (alpha = 0.05) are in italics; those in bold indicate higher proportion of individual case reports in the scale up compared to the pilot*.

*Data are from Guinea's national DHIS2, data for Pilot accessed in November 2017. Data for Scale up accessed July 2020*.

a *Diseases/events that are not mandated to be reported both in the weekly aggregate reports and in individual case reports were not included in the table (e.g., malaria)*.

b *Reporting on bloody diarrhea and cholera individual cases was changed to reporting on acute diarrheal syndrome when the individual case forms were revised in 2018*.

c *There were four sentinel sites for influenza-like illness in 2019. The sentinel sites are all in Conakry: Macire, Koulewondy, Communal Medical Center of Ratoma, and Gbessia Port 1. These sentinel sites are instructed to take samples from a five suspected cases per week and for any hospitalized cases, and to only complete a case notification form for cases that are sampled. For this reason, the denominator in this case is from the line list of sampled cases during the time frame rather than aggregate cases from DHIS 2*.

d *Reporting on viral hemorrhagic fever was changed to reporting on viral hemorrhagic fever syndrome when individual case forms were updated in 2018*.

During the RTI supervision visits, an observed challenge that contributed to delays, and possibly to missing case notification forms, was a lack of understanding of the procedures for entering individual case information into DHIS2. For example, some case forms were not entered into DHIS2 because the District Health Office was waiting for case identification numbers; however, the District Health Offices are in fact not supposed to enter case identification numbers into DHIS2, as the numbers are assigned at the national level. Another contributing factor was that some District Health Office staff had difficulty entering individual case information into DHIS2 Tracker due to its increased complexity (e.g., multiple questions and multi-part forms) compared to entering weekly aggregate report data.

Finally, it was observed that the health authorities at district, regional and national levels did not conduct active tracking and follow up on individual case reporting in DHIS2 during the pilot phase. This impacted the quality of the case report data in DHIS2, as there was little data cleaning to delete duplicates and follow up on missing information outside of project team-led supervision visits and communications. Factors that contributed to the missed follow up included lack of staff availability. Also, there were parallel reporting systems already in place during the pilot phase that limited health workers' time and interest in ensuring that individual case reports were entered in the DHIS2 Tracker.

As part of the evaluation of the pilot phase, a questionnaire was administered to participating personnel in the two pilot regions, Boké and Labé, in partnership with the MOH and partners (International Medical Corps, Alima, WHO, International Organization for Migration) in November-December 2017. The objective was to obtain users' feedback, evaluate their capacity to use the system, and better understand the challenges for national implementation. The questionnaire (provided in full in the Supplementary Material Data Sheet 2) included an assessment of skills in using DHIS2 for epidemiological surveillance for respondents at the district and regional levels, and questions about if and how they used DHIS2 for analysis of epidemiological surveillance data, their experience in entering individual case information in DHIS2, if their organization has adequate internet, electricity and functional computers, their participation in the pilot phase, recommendations for improving the training, if and how they have received laboratory reports for cases reported, data quality checking, and analysis of advantages and disadvantages of the new DHIS2 system. A total of 49 people completed the individual interviews and an assessment of DHIS 2 and basic computer troubleshooting at the district and regional levels (24 in Boké, 25 in Labé; see Supplementary Table 1).

All respondents agreed that the use of DHIS2 facilitated data analysis, and the majority also noted it facilitated data entry and data sharing. However, less than half of respondents cited the potential to receive laboratory results for reported cases as an advantage (Supplementary Table 2).

Participants achieved an average of 74% of correct responses on an assessment of basic computer troubleshooting skills and DHIS2 use (see Supplementary Table 3 for the list of assessment questions). On average users in Boké scored 68% on the assessment while Labé users scored 78%, although there were substantial differences in average scores across different job positions (Supplementary Table 4). Data managers were the largest category of users interviewed and assessed, and with an average of 90% correct responses, they scored the highest on average of all other groups except single users “Secretary” and “Head of Planning, Training and Research,” who both scored 100%. Laboratory Personnel and Clinicians scored the lowest, at 50 and 54% correct responses, respectively. Between regions, the largest difference in correct responses were those in Hospital Administrator/Supervisor positions (*n* = 8) with 44% correct responses for Boké and 67% for Labé.

The assessment results also revealed that users interviewed were least familiar with the individual case notification forms in DHIS2 (57%) as compared to other features and basic computer troubleshooting skills. The next lowest percentage of correct responses was for demonstrating the ability to use data analysis tools in DHIS2 to generate tables or graphs for aggregate weekly report data (61%). More respondents knew how to access tables, graphs and maps using the dashboard in DHIS2 (80%) (Supplementary Table 4).

A report on the pilot implementation was presented to stakeholders in the weekly meeting of disease surveillance stakeholders at the National Agency for Health Security in January 2018 and the main recommendations were validated through consensus. The recommendations, strengths and weaknesses noted from the evaluation of the pilot phase overall are summarized in Table 4.

**Table 4:** Summary of strengths, weaknesses, and recommendations from the evaluation of the pilot phase.

**Strengths**	**Weaknesses**	**Recommendations**
• Effective use of DHIS2 by the majority of trained people assessed • The availability of the infrastructure (electricity, internet) for the use of the tool • Participation of the national level and partners in evaluation activities • The availability of managers and users to improve their performance in DHIS2 • Support for users during the pilot phase	• Delay in sending units for telephones and internet • The non-use of the tool by all officials and stakeholders in certain districts • The non-effective use of data by the National Agency for Health Security data management unit • Failure to enter all suspected cases of epidemic-prone diseases or maternal deaths in the system • Failure to enter laboratory results in the laboratory section of the forms	• Enter all suspected epidemic-prone disease cases and maternal deaths into the system • Encourage effective use of the tool by the National Agency for Health Security data management unit • Extend DHIS2 training to all health structures (health centers) • Carry out regular supervision visits • Encourage use of the tool by all decision-makers at the district level • Increase training time • Find local trainers to start scaling up the tool • Extend training to all national actors • Follow up with all those who have been trained • Support the District and Regional Health Offices for effective use of the tool • Make sure the system is in French • Send cell phone/internet credits on time • Train / upgrade district officials and actors • Make the DHIS2 manual available

These recommendations were incorporated into the planning process for the scale up of DHIS2, as described in the following section. To begin planning for long-term sustainability, a dialogue was started with the University of Gamal Abdel Nasser of Conakry and the MOH to explore a partnership that would strengthen training of public health students in the use of DHIS2 for public health, and of computer science students and faculty in DHIS2 configuration and maintenance.

## Scale Up (January 2018-March 2020)

Here, we describe the first 2 years of the implementation and monitoring results for the scale up of DHIS 2 for epidemic-prone disease surveillance to all districts in Guinea, until the detection of coronavirus disease (COVID-19) in early March 2020.

### Scale Up of Aggregate Weekly Reporting

The scale up of DHIS2 for aggregate weekly disease surveillance began in January 2018 following validation of the pilot implementation evaluation by the National Agency for Health Security and other MOH stakeholders. One hundred and fifty-five hospital medical center directors, statistics managers, and head physicians in charge of disease surveillance from District Health Offices, and six Regional Health Offices, and District and Regional Hospitals in the remaining 28 of 38 districts not included in the pilot phase were trained on reporting and analysis of weekly aggregate surveillance data. The 10 health districts in the Boké and Labé regions that had already been trained during the pilot phase were not included. Four-day workshops were held in January 2018 in each region for the ~28 representatives from the District and Regional Health Offices in each region. Focal points for DHIS2 support were identified in each district and each region. During the scale up, the National Agency for Health Security required that all District Health Offices also continue to use the existing disease reporting system, based on an Excel template. Therefore, from January 2018 the District Health Offices were entering their data into two different systems.

### Scale Up of Individual Case Reporting

Individual case reporting in DHIS2 was delayed until the list of priority diseases was validated in August 2017, and the individual case reporting forms were revised and simplified (per the recommendations from the pilot) and configured in DHIS2 (see Table 1 for the list of epidemic-prone diseases and events included in DHIS2 2018-2020). The DHIS2 Tracker program is used for case notifications in DHIS2. From 2015 to 2020, each case notification form contained an epidemiological case identification that includes the country, region, health district, year, and a unique case number. This number is manually assigned after the form arrives at the national level. In 2021 there was an additional case identification variable added and it is assigned automatically when the case notification form is created in DHIS2. When the user registers a new case, the system alerts you when the information of the case is like the information of another already registered case. It is then up to the user to confirm if the case is different from the one(s) already registered before they can continue the registration. The laboratories use the same DHIS2 Tracker system to register receipt of laboratory samples and to enter test results. A copy of the case notification form accompanies each sample to the laboratory (see Figure 3 for a simplified data flow). When the sample arrives at the laboratory, the laboratory staff proceed to search for the case by searching for the health facility notifying the case and then entering the last name, first names and telephone number of the person responsible. When the case is found, the staff compare the age and names in the system with the information on the copy of the case notification form received at the lab. Despite these safeguards, it is still possible to enter the same case twice in the system and this reiterates the need for careful review of all data entered and correction of errors and duplications as quickly as possible.

From August 28 to September 3, 2018, a 6-day training of trainers' workshop was held for 68 representatives from the MOH, Ministry of Environment, Water and Forests, Ministry of Livestock, and partner representatives. The first 3 days were focused on an overview of the IDSR guidelines, revised case notification forms and job aids. The second 3 days focused on the use of DHIS2 for individual case notifications. Regional-level trainings for health officers at the District Health Office level were held following the same 6-day format from December 2018 to February 2019. In all, 388 people participated in the trainings. Following these trainings, trainees from District Health Offices, accompanied by national MOH staff and surveillance partners, rolled out the training to 1,269 health workers in 652 health facilities between April and May 2019.

### Scale-Up Results

In July 2019, the National Agency for Health Security deployed teams to each of Guinea's eight regions to work with the District Health Offices to address the lack of entry and late entry of individual case information in DHIS2, and to resolve discrepancies in the aggregate weekly reports between the Excel-based data and DHIS2 data. The teams were made up of personnel from the National Agency for Health Security, the MOH Office of Strategy and Development, the National Public Health Institute, and RTI International. The visits consisted of two-day workshops bringing together DHIS2 users including two individuals from each Regional Health Office and two individuals from each District Health Office (the data manager and the Chief Medical Officer in charge of disease surveillance). In total 88 people participated. Prior to the workshop, for each district, the facilitation teams identified discrepancies between the aggregate weekly disease surveillance reports in DHIS2 and in the Excel database. The discrepancies were presented, participants and facilitators were divided into health district groups to harmonize the data and enter missing information in the Tracker module of DHIS2. Finally, a training on the helpdesk platform was provided to allow users to report anomalies encountered in the use of the DHIS2 software and to receive assistance.

**Figure 4:**
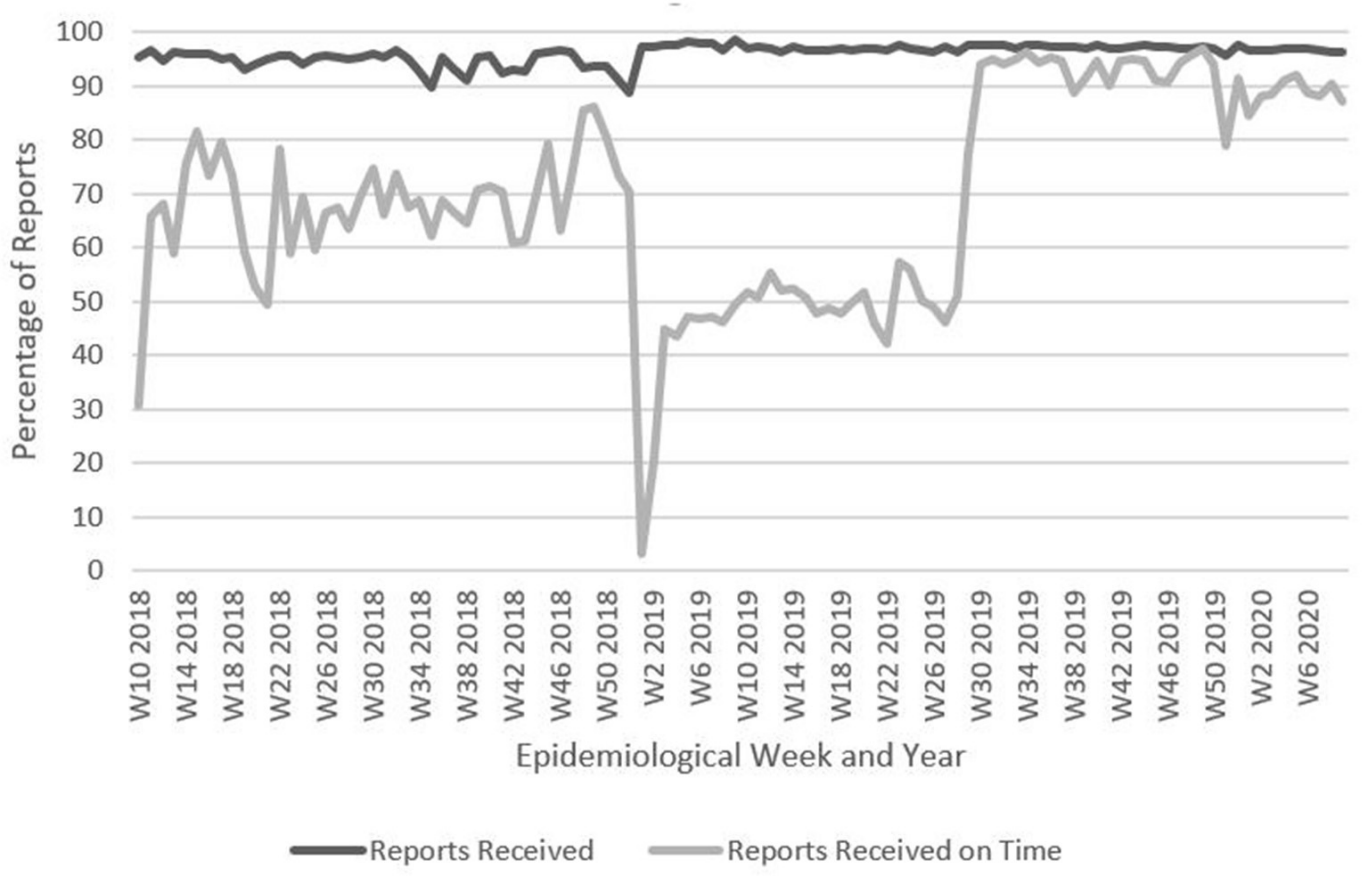
Percentage of weekly aggregate disease surveillance reports received and received on time, Guinea, Week 10, 2018–Week 10, 2020. Data are from Guinea's national DHIS2: https://dhis2.sante.gov.gn/dhis/dhis-web-commons/security/login.action which has changed to https://entrepot.sante.gov.gn/dhis. The data for reports received on time for weeks 1–9, 2018 are missing. Data for reports received from weeks 1–9, 2018 is not significantly different than for other weeks of the year therefore these weeks are not included.

Completeness and timeliness for weekly aggregate disease reports and individual case reporting in DHIS2 were analyzed as for the pilot implementation, from epidemiological week 26, 2018 to week 10, 2020 (Figure 4). Additionally, in Boké and Labé, data on completeness and timeliness between the pilot implementation and scale up phases were also compared.

The completeness of reporting was largely similar in the pilot phase and scale up phase. The timeliness of reporting was slightly higher for the pilot phase (see Table 5).

**Table 5:** Average timeliness and completeness of aggregate disease surveillance reports in DHIS2 during pilot and scale up phases.

**Measure**	**Pilot (weeks 22–43, 2017) (10 Districts)**	**Scale up (week 26, 2018–week 10, 2020)** **(38 Districts)**
Average timeliness	82.7%	71.3%
Average completeness	99.5%	96.6%

*Data are from Guinea's national DHIS2. For “Pilot,” data were accessed November 2017; for “Scale up” June 2020*.

To assess the extent to which users were completing and entering individual case information in the DHIS2 Tracker after all health facilities had been trained on and received the new forms, cases reported in aggregate reports were compared with individual case reports in the Tracker (see Table 3). The data comparing the number of individual case reports against the aggregated total number of weekly cases show that during both the pilot and scale up phases there was a wide gap between the number of cases reported in the weekly aggregate report and the number of individual case reports entered into DHIS2, across most diseases. This is particularly striking for influenza-like illness, where even though the denominator is restricted to those cases which are sampled for laboratory confirmation (maximum of five suspected cases per week, plus any hospitalized cases), only 22% of individual case reports for influenza-like illness were submitted compared to the total line list number of sampled cases. Differences between the proportions of individual case forms and aggregate numbers during the pilot and scale up were calculated using Chi-squared tests. Acute flaccid paralysis and measles individual case reports as a proportion of aggregate cases dropped significantly between the pilot and the scale up. One potential reason for lower number of individual case reports vs. aggregate case numbers, particularly for diseases like measles that frequently cause outbreaks in Guinea, may be that under the prior manual reporting system, once a certain number of cases of a disease had been detected, District Health Offices were no longer required to report individual cases, but rather summary line lists. District health officials may be continuing to follow this approach in DHIS2, suggesting the need for clarification of guidance on case reporting during outbreak situations. If this is the case, larger numbers of overall cases during the scale period up might account for the lower relative proportion of individual case forms during the scale up, if more outbreaks occurred during that time or across different districts. Conversely, rabies individual case reports vastly exceeded aggregate reported cases; follow-up revealed that District and Regional health personnel were using the rabies case reporting form to report any type of animal bite, including snakebite, thus inflating the individual case reports to 276 compared to nine aggregate reported cases of rabies. Neonatal/maternal tetanus individual case reporting increased significantly between the pilot phase and the scale up, a promising sign of acceptance of the overall approach as training on the forms was rolled out, especially for less common diseases.

We used individual case data on measles to analyze timeliness, calculated as the average number of days between the patient consultation date and date of data entry in DHIS2. District Health Offices are expected to enter case notifications from their constituent health facilities in DHIS2 as they are received. The job aids for IDSR developed in 2018 and disseminated to all health facilities as well as Regional and District Health Offices in 2019 outlined that the health facility reporting a case should send the laboratory sample and two copies of the case notification form to the District Health Office within 24 hours of the case notification and the District Health Office should enter case notification forms into DHIS2 within 24 hours of receiving the hard copy of the notification form from the health facility. Measles was selected due to having the highest number of suspect cases in 2019 among all epidemic-prone diseases reported by individual notification form.

**Figure 5:**
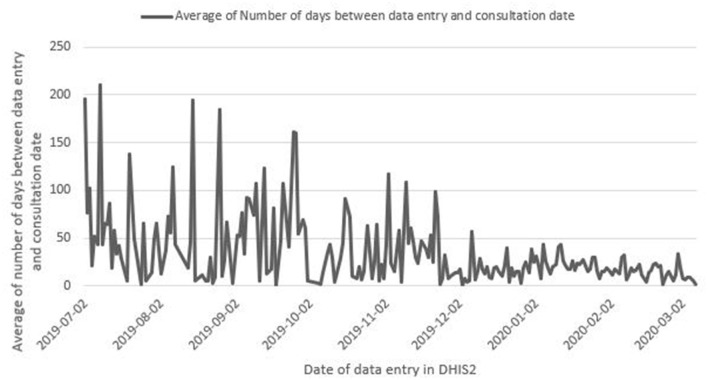
Average number of days between consultation date of the patient and data entry in DHIS2 for measles case notifications, Guinea, June 30, 2019-March 7, 2020. Data are from Guinea's national DHIS2, accessed June 10, 2020.

There was substantial variation in the time between patient consultation and data entry of the individual case report form in DHIS2 for measles, with an average delay of 22.9 days (s.d. = 30.2) (*n* = 2,027) (Figure 5). Follow-up investigations, conducted retrospectively through discussions with staff at the National Health Security Agency suggest that some of the higher numbers of days between consultation date and data entry data from December 4 – 11, 2019 may be caused by back-entry of older case notification forms into DHIS 2 by districts at the request of the National Agency for Health Security. Figure 5 shows there was an overall decrease in the number of days between consultation and entry of case notifications in DHIS2 over time during the scale up phase.

### Linkages to FETP and Local University

To strengthen DHIS2 implementation for disease surveillance, its use was included in the Field Epidemiology Training Program (FETP). In addition to being taught about DHIS2, participants are required to use the system in their daily work and FETP assignments. For example, one of the assignments of the FETP-Frontline is to produce a weekly epidemiological bulletin using the data from DHIS2. Similarly, one of the assignments of the FETP-Intermediate is to analyze a 5-year database for a specific disease. Students were required to identify all available data for their chosen disease, consolidate them in DHIS2 if not already done, and use the DHIS2 database for the analysis.

In addition, an agreement was signed in August 2018 to formalize a partnership between RTI and the Gamal Abdel Nasser University of Conakry to train Master of Public Health students in DHIS2, and computer science faculty and students on its configuration and maintenance to enhance the long-term sustainability of the platform. The partnership included support to refurbish two university computer labs and provide internet access. Computer science faculty participated in DHIS2 trainings, including workshops on DHIS2 server management and configuration. Overall, the partnership enhanced the capacity of the university to support the MOH with DHIS2 maintenance and use, although further evaluations will be needed to determine the extent of the impact of this initiative on long-term sustainability.

## Lessons Learned and Recommendations

**Figure 6:**
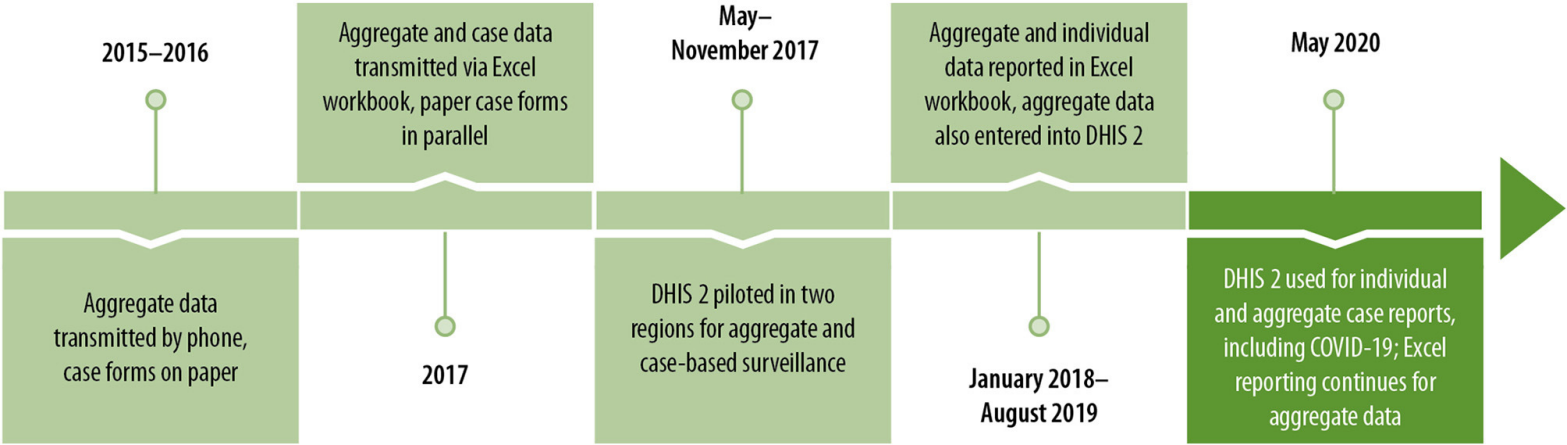
Evolution of disease surveillance in Guinea, 2015–2020.

Since 2015, Guinea's epidemic-prone disease surveillance reporting has evolved substantially, from a system focused on aggregate reporting only, transmitted from health facilities to the national level by phone and case forms on paper, to a fully integrated and electronic disease surveillance system that allows both for aggregate and individual case reports, and which can quickly be adapted to include emerging disease threats such as COVID-19 (Figure 6).

### Factors Influencing the Timeframe for Development and Scale Up

This extended process of enhancing the surveillance system through implementation of DHIS2 uncovered some lessons learned and recommendations related to improving the adoption of digital health platforms (Supplementary Table 5) and health systems strengthening broadly.

While the adoption of DHIS2 as the national health information system platform, including configuration, piloting and scale up for routine monthly health reporting from facilities, was relatively short (about 1 year), adopting DHIS2 for disease surveillance took substantially longer. The process of configuration, piloting, scale up and decision to discontinue parallel surveillance and reporting in Excel took a ~3 years. The switch-over from Excel has still not been completed as of January 2021, despite suggestions that completeness of data entry in DHIS2 may be negatively impacted by data managers' attention being split across the two data formats. The slower adoption of DHIS2 for disease surveillance could be attributed to several factors. One was the hesitation of the National Agency for Health Security leadership to adopt a new system due to poor experiences with prior information systems, including losses of data and system failures. To prevent data loss and promote security several strategies were used: 1) access to the system was controlled by the Ministry of Health, you had to request access to the system through the National Health Information System office and after approval you are only given access to data or permission to edit data based on your role; 2) the system was hosted at the Guinea Broadband (GUILAB) facility, a secure location in the country with availability of 24 x 7 electricity and internet access; 3) the DHIS2 software is thoroughly tested and we used a version that was in production long enough to be stable and have most bugs addressed; and 4) database backups were done automatically every day, every week and every month at 00:00. At the end of the execution of the backup script, an email was sent to the pool of administrators to inform them whether the task was successful or not. These backups were stored in the server. To avoid filling the server only the daily backups of the last week, the last 2 weeks and the last month were saved.

Another factor was the West Africa Ebola outbreak, which preoccupied disease surveillance stakeholders until the outbreak was officially declared over in June 2016. Additionally, during this time, the MOH and stakeholders decided that the priority diseases/events under surveillance needed to be expanded, and that all case notification forms needed to be harmonized and updated before implementing individual case reporting in the DHIS2 Tracker. This process took over a year and required training 652 health facilities nationwide, from the national to the sub-district level, and distributing copies of the new forms. At times, implementation activities were delayed due to competing priorities of the MOH and the National Agency for Health Security, and lack of adequate personnel to cover all activities. Administrative and communication challenges associated with the funding for the work, and specifically the implementation of trainings and printing of the case forms, also contributed to delays. Finally, the deliberate inclusion of all stakeholders, while critically important for reaching consensus and ensuring buy-in, contributed to the duration of the process.

### Challenges Related to Individual Case Notification

Overall, several of the challenges observed in implementing the DHIS2 for electronic notification of individual cases mirrored the challenges to the collection of the data using the previous paper-based reporting system. One of the major problems observed was that district and sub-district facilities did not complete individual forms for all cases notified through the weekly aggregate disease reports. Possible reasons may include lack of: (1) adequate human resources to complete forms especially where there is a high volume of cases; (2) use of data from case forms and supervision to ensure the use of case forms to notify all cases; (3) understanding of the requirement to use case forms to notify all cases; and (4) feedback from the national to the district level on the data reported. The pilot and scale up phases demonstrated the importance of regular MOH follow-up with the District Health Offices to ensure that all cases are reported using the individual case notification form in DHIS2 in a timely manner. There are currently plans to train staff from over 1,000 public and private health centers on DHIS2, to encourage the entry of data into DHIS2 at the sub-district and local levels, and alleviate the burden experienced by district data managers. These efforts, which will be carried out in 2021, will also include training of “super-users” at the national and regional levels, as well as lab technicians and data managers associated with the national immunization program. Simultaneously, there will be a push to phase out Excel-based reporting.

### Leveraging DHIS2 for the COVID-19 Response

DHIS2 provides a user-friendly and extremely adaptable interface for health information systems and can also be specifically tailored to higher resolution disease surveillance. Advantages of DHIS2 include a large international community of users that can provide technical support, access to new modules and add-ons, and sharing of experiences across countries, regular software updates, availability of documentation and in-person and online training resources, and no software licensing fees. In addition, in countries using DHIS2 for the routine health information, the use of it for disease surveillance helps improve the sustainability of the overall system and eliminates the need to maintain parallel systems. The influx of resources to Guinea for the Ebola response helped lay the foundation for the successful implementation of DHIS2, as the MOH was able to provide computers and solar power kits to all of its health facilities and offices and conduct basic computer skills training for personnel. The West Africa Ebola outbreak made urgently clear the need for real-time surveillance systems and provided resources to help countries like Guinea enhance surveillance. In Guinea, COVID-19 has motivated stakeholders to further adopt DHIS2 as an integral structure to respond to this pandemic, and indeed, the National Agency for Health Security swiftly established a COVID-19 module for case data in March 2020. Using DHIS2 for the COVID-19 response, stakeholders, including laboratories, now better understand and support the process of individual case reporting and timely data entry as they can see the line lists with case characteristics, produce summary statistics, and have a real-time dashboard that can be used for evidence-base decision making. The availability of the already established DHIS2 platform for surveillance, quickly adapted for COVID-19 response, suggests that investments made as a result of the West Africa Ebola outbreak have enhanced the national and local health systems capacity to response to the COVID-19 pandemic. It will be important to analyze the performance of DHIS2, and specifically the completeness and timeliness of both COVID-19 and non-COVID-19 disease surveillance data, to determine whether these systems are resilient despite the added pressure caused by the pandemic.

### Needs for Workforce Development and Supervision

The implementation of DHIS2 for disease surveillance is a significant and transformational undertaking—-it requires substantial political will, across different levels of government (national and sub-national) and different parts of MOH (lab, health information systems program, vertical programs). However, a health information system is only as useful as the data within it, and how these data are used. Much training and retraining is needed to support staff at all levels to identify and report cases of epidemic-prone diseases in a timely and accurate manner, to enter data into DHIS2 on time, to monitor data quality and correct issues, and to analyze and use the data effectively. Efforts such as training on IDSR and integration with FETP can substantially increase the quality of surveillance data, and provide a platform for improving other factors relevant to timely and responsive disease surveillance. In this way, DHIS2 works hand in hand with workforce development initiatives to provide easier and more reliable access to actionable data that can be used at national, regional and district levels for monitoring, investigation, and response.

### Sustainability

Finally, a critical observation relates to approaches for ensuring sustainability of the DHIS2 platform. A good strategy is to integrate the development of local capacity in DHIS2 configuration, management, and technical support into the implementation process, such as through university partnerships and incorporation in existing training programs such as FETP. FETP graduates' knowledge and use of DHIS2 enables them to continue to reinforce DHIS2 from within the system. The partnership between the Gamal University and MOH followed a model that has been successful in other countries provides technical support for DHIS2 and creates a pipeline of expertise in support of maintaining the system. While the integration of DHIS2 in FETP was easy, the implementation of the university partnership in Guinea was not without challenges. There was a long delay in obtaining official approval of the university partnership from the MOH, and challenges in finding ways to engage the computer science faculty and students in the DHIS2 work. Finally, there were challenges in ensuring the availability of required physical resources to enable effective use of the system including internet access, electricity, training, supervision and user support, server maintenance and hosting, skilled staff to maintain the system and to strengthen data quality and use. Finally, Guinea's dependence on donors for routine costs of its disease surveillance system leaves the sustainability of this national system in the hands of partners. However, the large initial investment can be countered by relatively low recurrent costs—-mainly related to maintenance (of infrastructure, internet connectivity, software upgrades, etc.), supervision, and refresher trainings. It is important to plan from the outset how these recurring costs can be transferred to the MOH in the long-term. Cost analyses have not yet been performed and will be important to undertake in the future to ensure appropriate planning for recurrent costs and to help understand the costs and benefits of the system. In Guinea, a transition plan was developed between partners and the MOH, which focused on ensuring that the MOH staff were trained and mentored and ready to assume responsibility for the management of the system.

Forty-two countries are using DHIS2 for COVID-19 disease surveillance including 18 in Africa^7^. In Guinea, a local team trained on DHIS2 during the post-Ebola recovery period configured the system and set up the COVID-19 dashboard for the National Agency for Health Security quickly without international assistance. This transfer of expertise and systems improvement provides much encouragement around countries ability to adopt, sustain, and improve DHIS2 as a critical component of their epidemic-prone disease surveillance and reporting systems to enhance compliance with the IHR.

## Data Availability Statement

The data analyzed in this study is subject to the following licenses/restrictions: Data from Guinea's Ministry of Health is non-public data, however data used in the analyses can be shared upon request. Data on project activities can be shared upon request. Requests to access these datasets should be directed to ereynolds@rti.org.

## Author Contributions

ER developed the original concept for the article, contributed to analysis of the data, wrote and revised the article, provided management, and supervision of the activities reflected in the article. LM contributed to the development of the article, reviewed drafts, and provided substantial feedback on the article structure and content. CS contributed to the analysis of the data and writing the article. PM provided direction for the project (reflected in the article), provided direction for the concept and contributed to writing the article. ER, LM, PM, MOB, MB, MBB, BB, NC, YC, BD, ID, MKD, MTD, TD, SG, JH-F, FH, AK, AKK, MK, DL, KM, NS, OS, KS, ST, and MW implemented the project and activities on which the article draws. DL and SC reviewed data and analyses used in the manuscript. YC, ID, and AK provided access to data from Guinea's national DHIS2 system to enable the data analyses in the manuscript. All authors contributed to the review and approval of the final manuscript.

## Funding

This publication was supported by Cooperative Agreement U19GH001591 funded by the U.S. Centers for Disease Control and Prevention.

## Author Disclaimer

Its contents are solely the responsibility of the authors and do not necessarily represent the official views of the Centers for Disease Control and Prevention or the Department of Health and Human Services.

## Conflict of Interest

The authors declare that the research was conducted in the absence of any commercial or financial relationships that could be construed as a potential conflict of interest.

## Publisher's Note

All claims expressed in this article are solely those of the authors and do not necessarily represent those of their affiliated organizations, or those of the publisher, the editors and the reviewers. Any product that may be evaluated in this article, or claim that may be made by its manufacturer, is not guaranteed or endorsed by the publisher.

## Footnotes

^1^ https://dhis2.org/integration/

^2^Participants included representatives from the National Agency for Health Security; National Health Information System office (part of the Office of Strategy and Development in the MOH), Directorate for Disease Prevention and Control, Expanded Program on Immunization, WHO, CDC, RTI International, Regional and District Health Offices, National Malaria Control Program, Directorate of Family Health and Nutrition, International Organization for Migration, International Medical Corps, health centers (sub-district level), and two national reference laboratories: the National Public Health Institute and the Hemorrhagic Fever and Virology Laboratory.

^3^Representatives from each MOH pyramid level, the ANSS, WHO, RTI, CDC, and AFENET.

^4^Not included in weekly aggregate report.

^5^Other diseases such as malaria are reported in aggregate weekly reports but do not use individual case report forms.

^6^There was also sentinel surveillance for influenza-like illnesses with a sample of cases reported individually and laboratory testing conducted.

^7^https://dhis2.org/in-action/ (accessed on December 7, 2021).
