# Supplementary material for: Implementation of DHIS2 for Disease Surveillance in Guinea: 2015–2020
Source: Front Public Health. 2022 Jan 20;9:761196. doi: 10.3389/fpubh.2021.761196 (PMC8811041; doi:10.3389/fpubh.2021.761196)
Supplement: Supplementary file 1 [file Table_1.docx]

**Supplement 3. Pilot Phase Questionnaire Respondents by Job Function and Region**

Table S1. Pilot Phase Questionnaire Respondents by Job Function and Region

| Region | Job Function | Number of People Interviewed |
| --- | --- | --- |
| Boké | District Health Director | 4 |
|  | Data Manager | 3 |
|  | Laboratory worker | 3 |
|  | District Chief Medical Officer | 4 |
|  | PFR | 1 |
|  | Statistician | 3 |
|  | District Hospital Director | 3 |
|  | Secretary | 1 |
|  | Centre for Treatment of Epidemic Prone diseases health worker | 2 |
|  | Sub-total | 24 |
| Labé | District Health Director | 4 |
|  | Data Manager | 5 |
|  | Hospital manager | 4 |
|  | District Chief Medical Officer | 2 |
|  | Regional Chief Medical Officer | 1 |
|  | Statistician | 7 |
|  | Regional Health Director | 1 |
|  | Data entry | 1 |
|  | Sub-total | 25 |
|  | **Grand Total** | **49** |
