# Supplementary material for: Implementation of DHIS2 for Disease Surveillance in Guinea: 2015–2020
Source: Front Public Health. 2022 Jan 20;9:761196. doi: 10.3389/fpubh.2021.761196 (PMC8811041; doi:10.3389/fpubh.2021.761196)
Supplement: Supplementary file 2 [file Table_2.docx]

**Supplement 4: Table S2. Percentage of Users that agreed with specific advantages of DHIS2**

Table S2. Percentage of Users that agreed with specific advantages of DHIS2

| **Region** | **Facilitates data analysis** | **Facilitates data entry** | **Facilitates data sharing** | **Allows you to receive laboratory results** | **Allows you to compare data** |
| --- | --- | --- | --- | --- | --- |
| **Boké** | 100% | 92% | 88% | 50% | 71% |
| **Labé** | 100% | 76% | 84% | 28% | 48% |
| **National level** | 100% | 80% | 80% | 60% | 60% |
