# Supplementary material for: Implementation of DHIS2 for Disease Surveillance in Guinea: 2015–2020
Source: Front Public Health. 2022 Jan 20;9:761196. doi: 10.3389/fpubh.2021.761196 (PMC8811041; doi:10.3389/fpubh.2021.761196)
Supplement: Supplementary file 3 [file Table_3.docx]

**Supplement 5: Table S3. Positive responses on DHIS 2 and basic computer troubleshooting skills assessment questions**

Table S3. Positive responses on DHIS 2 and basic computer troubleshooting skills assessment questions

| **Question** | **Affirmative Responses**  **N=49** | **%** |
| --- | --- | --- |
| If your internet credits are used up, do you know how to activate your credits and verify your consumption? | 45 | 92% |
| Do you know the difference between the « complete » button and the « incomplete » button?^[[1]](#footnote-1)^ | 44 | 90% |
| Have you entered or verified data in the DHIS 2? If yes, can you show me an example? | 43 | 88% |
| Do you know how to visualize the dashboard in DHIS 2? If yes, can you show me an example? | 39 | 80% |
| Do you know how to change your browser in case of problems? | 38 | 78% |
| Have you entered or verified weekly SAP data in the DHIS 2? If yes, can you show me an example? | 36 | 73% |
| Have you entered or verified maternal death notification forms in the DHIS 2? If yes, can you show me an example? | 34 | 69% |
| Can you write a message in DHIS 2 and send to other users? | 33 | 67% |
| Can you save a chart/graph/map/table as a favorite in a dashboard you have created? | 32 | 65% |
| Can you explain how you generate a report on data completeness? | 31 | 63% |
| Can you do a demonstration of how to use the data analysis tools for SAP data? | 30 | 61% |
| Have you entered or verified individual case notification forms in the DHIS 2? If yes, can you show an example? | 28 | 57% |

1. These buttons appear on the data entry screens in DHIS 2, selecting the “complete” button indicates to the system that data entry for that period is complete. [↑](#footnote-ref-1)
