# Supplementary material for: Implementation of DHIS2 for Disease Surveillance in Guinea: 2015–2020
Source: Front Public Health. 2022 Jan 20;9:761196. doi: 10.3389/fpubh.2021.761196 (PMC8811041; doi:10.3389/fpubh.2021.761196)
Supplement: Supplementary file 4 [file Table_4.docx]

**Supplement 6: Table S4. Average Correct Responses to DHIS 2 and Basic Computer Troubleshooting Skills Assessment by Position**

Table S4. Average Correct Responses to DHIS 2 and Basic Computer Troubleshooting Skills Assessment by Position

| **Position** | **% of Users Assessed** | **Count** | **Percentage correct** |
| --- | --- | --- | --- |
| Secretary | 2% | 1 | 100% |
| Head of Planning, Training and Research | 2% | 1 | 100% |
| Data Manager | 39% | 19 | 90% |
| District Health Director | 16% | 8 | 67% |
| Chief Medical Officer | 14% | 7 | 62% |
| Hospital Administrator/Supervisor | 16% | 8 | 58% |
| Clinician | 4% | 2 | 54% |
| Laboratory Personnel | 6% | 3 | 50% |
| **Grand Total** | **100%** | **49** | **74%** |
