# Supplementary material for: Implementation of DHIS2 for Disease Surveillance in Guinea: 2015–2020
Source: Front Public Health. 2022 Jan 20;9:761196. doi: 10.3389/fpubh.2021.761196 (PMC8811041; doi:10.3389/fpubh.2021.761196)
Supplement: Supplementary file 5 [file Table_5.docx]

**Supplement 7**

**Table 5**: **Lessons Learned from the Planning and Adoption, Piloting and Scale up of DHIS2 as a Digital Health Platform for Disease Surveillance and Recommendations for Future Efforts in Guinea and Other Similar Contexts**

| **Phase of Project** | **Challenge Observed** | **Lessons Learned** | **Recommendations** |
| --- | --- | --- | --- |
| **Planning & Adoption** | Stakeholder hesitancy | Stakeholders’ past experiences, and particularly negative ones, in digital health systems projects must be taken into consideration, and trust built progressively, to gain buy-in. | Providing demonstrations of strong data security and back-ups as part of the socialization of the project may prove useful, to assuage common concerns related to using digital health platforms for sensitive data. |
|  | Stakeholder understanding | Successful adoption is predicated on stakeholders’ understanding the added value of DHIS2 for disease surveillance. | On-going disease outbreaks, or even analysis of routine data, can be used for direct demonstrations to key stakeholders of how the dashboards and data analysis functions in DHIS2 can be used with in-coming data to support response efforts or routine management of surveillance teams. |
|  | Ensuring MOH buy-in and leadership | MOH leadership is fundamental to encourage adoption of DHIS2. Without such leadership, departments, district/regional offices, and programs within the MOH are unlikely to change their current systems. Even with instruction from the MOH that DHIS2 was to be used as the sole information system, it took a lot of effort to gain buy in for the transition to its use for disease surveillance. | Socialization and information-sharing must be a continuous and sustained process throughout the project and must be effected at multiple levels of administration – from top decision makers in the MOH down to the end users at the sub-district level. |
| **Implementation** | Lack of case notification data quality and completeness | A stronger focus on data use from the beginning of the forms development (ensuring that all data collected would be useful and used) would likely have contributed to greater demand for case forms to be completed fully and correctly and entered into DHIS2 in a timely manner so that the data could be analyzed and used. It may have also led to streamlining of notification forms to only the most essential data, reducing burden on health workers, speeding up data entry/reporting, and improving data quality. | Ensuring that a strategy for data use is developed and socialized alongside implementation of the DHIS2 system will strengthen the data quality. This could include development of customized “dashboards” for different MOH programs or other end-users, tailored to their specific needs and priorities. |
|  | Lack of updated guidelines for entry of case notification data into DHIS2 during an outbreak | The process of scale up of DHIS2 for individual case notifications revealed issues with lack of guidance regarding when to complete the full individual case form versus switching to line list counts during disease outbreaks. | Detailed guidance on reporting during outbreaks should be developed, disseminated, and implemented when introducing the use of a new system for individual case notifications, to ensure the completeness and quality of the data. This guidance would help improve data quality and reduce reporting burden on health facilities in times of elevated cases. |
|  | Lack of completeness and timeliness of laboratory reporting in DHIS2 | Laboratories were slow to enter test results and other data into DHIS2 during the pilot and the initial scale up. The laboratories have multiple databases into which they must enter data and are thus experiencing the burden of multiple parallel reporting systems. | Strong engagement and demonstration of the benefits of DHIS2 to the laboratories can facilitate quicker adoption and use of the new system. Bringing stakeholders together to find ways to reduce parallel reporting through information sharing and harmonization or systems interoperability can be used to strengthen the timeliness and completeness of laboratory results reporting in DHIS2. Building in dedicated positions and funding for data management staff can improve data quality and avoid hindering other aspects of the response through overburdened health personnel. |
|  | Parallel reporting systems for disease surveillance | The extended period of parallel reporting in Excel and DHIS2 led to many challenges including discrepancies in the data between the two systems, leading to doubts of DHIS2 readiness to be adopted, reporting burdens for District Health Offices, and difficulty in making progress towards adoption of DHIS2. | Parallel reporting periods can be shortened through careful planning to progressively transition system functions, and through cultivation of trust of decision-makers through evidence and demonstrations of DHIS2 capabilities.  Training local level (i.e. health center) staff to conduct data entry in DHIS2 may also help alleviate data entry burdens on personnel at higher levels of the health system (i.e. district). |
|  | Lack of completeness of case notifications for certain diseases in DHIS2 | While the timeliness of the availability of individual case data for measles in DHIS2 increased over time during the scale-up phase, this represents only 33% of overall measles cases reported in aggregate reports. This indicates that there remain challenges to strengthening the availability of this data. | Shortening case notification forms, increasing health facilities’ ability to report cases directly in DHIS2 Tracker, clear policies on when to revert to limited data collection/line-listing (vs. filling out the entire case form) in DHIS2, and elimination of parallel individual case reporting (i.e. in Excel) could help strengthen the timeliness of the availability of individual case data in DHIS2. |
|  | Lack of adequate data quality review for case data entered in DHIS2, lack of understanding by health workers of procedure for reporting certain events | Data consistency and quality was variable. In some cases, it was observed that individual case reports in DHIS greatly exceeded weekly aggregate case counts (i.e. for rabies). These were later attributed to health workers’ use of the case forms to report other related illnesses or events (i.e. the rabies case form was used for dog, cat and snake bites). | District-level health offices need to carefully review cases reported and provide guidance to health facilities on the use of case forms. Intermediate health offices and national level authorities need to monitor and provide guidance to the districts if they fail to catch or correct such issues. Health workers may become overstretched during outbreaks, increasing likelihood of data inconsistencies. Systems could be put in place to enhance data monitoring, or provide additional checks, to maintain data quality even when staff are focused on other priority health issues. |
| **Overall** | Lack of adequate local technical capacity in DHIS2 to maintain the system long term | The project invested substantial resources in the training of local project, MOH and university staff to help ensure capacity was available for long-term maintenance and upkeep of the system. The formal training was done through DHIS2 Academies, and through attendance in workshops to set up the initial DHIS2 in Guinea. A training on DHIS2 was also incorporated into the FETP to strengthen capacity of trainees. | Building strong local technical capacity in DHIS2 helps long term sustainability by leaving in place local professionals who can provide support beyond the life of the project. Partners should invest in longer term work to engage universities and other local organizations who can provide additional technical capacity in sustaining investments in information systems. Leveraging regional and international opportunities for continued training can also help build networks of expertise and opportunities for peer-to-peer troubleshooting when issues arise. |
|  | Weak linkages between FETP and DHIS2 initiatives | Stronger linkages of the DHIS2 implementation with the FETP training program could have helped accelerate the process of adoption, as the FETP curriculum addresses data management, analysis and use capacity building, and involves many of the same individuals responsible for managing epidemic-prone disease data at the District level. | DHIS2 implementation efforts should work hand in hand with FETP, where such programs are in place, to incorporate DHIS2 into the FETP and engage trainees in active use of the system and in assisting other users in the field. The FETP mentor program should also incorporate the training and use of DHIS2 so that mentors can assist trainees in the field and reinforce what they learn in their training. |
