# Supplementary material for: Implementation of DHIS2 for Disease Surveillance in Guinea: 2015–2020
Source: Front Public Health. 2022 Jan 20;9:761196. doi: 10.3389/fpubh.2021.761196 (PMC8811041; doi:10.3389/fpubh.2021.761196)
Supplement: Supplementary file 6 [file Data_Sheet_1.docx]

**Supplement 1: Information Technology Infrastructure Assessment Criteria**

The Information Technology infrastructure assessment was conducted by RTI and Ministry of Health staff in November 2015 and covered National level offices, Regional and District Health Offices and hospitals to assess readiness to use DHIS 2 for the national epidemic prone disease surveillance system. During the assessment the following criteria were used:

Internet

- Is there a professional internet connection? (defined as internet connection with a guaranteed minimum speed including fiber optic, WiMax, or ADSL)
- What is the quality of the professional internet connection? To assess the quality of the connection, we use the ratio of the speed to the number of users.
- Which entity provides funding for the professional internet connection?
- Is there 3G internet coverage?
- What is the quality of the 3G coverage?
- Which entity provides funding for the 3G coverage?

Equipment: We counted only functioning equipment.

- Number of computers
- Number of printers
- Number of scanners
- Number of tablets
- Number of 3G modems

Governance – Finance

- Monthly cost of the Internet connection
- Are there procedures for the management of IT infrastructure including the Internet, network security, IT maintenance and loss of equipment?
- Is there a data backup management procedure?
- Contribution of development partners for the IT infrastructure (Guinean Francs)
- Contribution of the Guinean government for internet costs (Guinean Francs) (Does your management have a budget line to cover Internet costs?)

Security and Maintenance

- Is access to the main building secure?
- Number of computers over 3 years old
- Is there a licensed anti-virus software?
- Are there internal or external personnel to provide equipment maintenance?
- Is the person responsible for IT maintenance internal to the office or external?
- Do you regularly back up data to external drive / other physical location / cloud? If so, what type of backup storage is used?

Users

- Number of staff in your office
- Number of data management staff in your office
- Basic computer skills of your data management staff:
  - MS Word
  - MS Excel
  - MS Access
  - Internet/Email
  - Computer maintenance

Energy Sources

- Do you have a generator?
- Do you have solar panels?
- Do you have power from the Guinea Electricity grid?
- What is the average number of hours per day that you have electricity?
