# Supplementary material for: Implementation of DHIS2 for Disease Surveillance in Guinea: 2015–2020
Source: Front Public Health. 2022 Jan 20;9:761196. doi: 10.3389/fpubh.2021.761196 (PMC8811041; doi:10.3389/fpubh.2021.761196)
Supplement: Supplementary file 7 [file Data_Sheet_2.docx]

**Supplement 2 : Questionnaires for Central Level and Regional and District Level Health Personnel, Evaluation of DHIS 2 Pilot Test**

**1. Questionnaire for Central Level Health Personnel**

**FICHE DE COLLECTE DES DONNÉES SUR LA PHASE TEST DU DHIS 2 GUINEE**

**QUESTIONNAIRE A UTILISER AU NIVEAU CENTRAL**

Numéro du questionnaire: [__|___|__]___]

Informations personnelles

Nº [__|___|__]

Date de l’enquête: [__|__] /[__|__]/[__|__]

Mon nom est…………………………………………au nom du Ministère de la Santé en collaboration avec RTI, nous sommes en train de collecter des informations pour évaluer les activités du DHIS 2. Nous vous prions de répondre aux questions afin de nous aider à mieux comprendre les acquis et les besoins d’améliorations à apporter avant la mise à l’échelle du module dans le reste du pays.

Vos réponses seront tenues strictement confidentielles. Votre nom ne sera pas inscrit sur le questionnaire et aucun lien ne pourra être fait entre ce que vous me dites et votre nom. Vous n’êtes pas obligé(e) de répondre à une question et vous pouvez arrêter l’entretien à tout moment. Votre participation est volontaire et cet entretien ne durera pas plus de 60 minutes.

Je certifie que l’enquêté(e) a été informé(e) de la nature, du but de l’évaluation et qu’il (elle) a donné son consentement pour participer dans cette évaluation.

Signature de l’enquêtée : ___________________________________________

Signature de l’enquêteur : ___________________________________________

Date de la signature de la fiche d’enquête par l’enquêteur : [__|__] /[__|__]/[__|__]

*A TOUS LES ENQUETEURS : VOUS VEILEREZ A ENCERCLER, COCHER ET/OU INSCRIRE LA REPONSE DES REPONDANTS A LA PLACE PREVUE A CET EFFET.*

**INFORMATIONS GENERALES SUR LA PERSONNE INTERVIEWEE AU NIVEAU CENTRAL**

1. Structure: ..................................................................................................................................
2. Poste occupé dans la structure: ...............................................................................................
3. Date d’entretien : .....................................................................................................................

| **Questions et filtres** | | **Réponses** | | | | |  |
| --- | --- | --- | --- | --- | --- | --- | --- |
| 1. **Analyse des données** | | | | | | |  |
| 1. Est-ce que vous savez analyser les données de surveillance épidémiologique dans le DHIS 2 ? | | Oui | | Non | | | Pas de réponse |
|  |  | 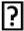 | | 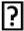 | | | 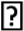 |
| 2. Si Oui, quels modules dans le DHIS 2 avez-vous utilisé pour les analyse de données ? (Cocher toutes les réponses mentionnées par le répondant.  **Ne lisez pas les propositions de réponses** | | tableau  croisé  dynamique | | Système d’Information Géographique | | | visualiseur de données |
|  |  | 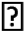 | | 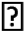 | | | 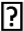 |
|  |  | rapports d’évènements | | rapports standards | | | Visualiseur d’évènement |
|  |  | 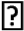 | | 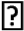 | | | 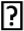 |
| 3. Si oui avez-vous partagé le résultat de vos analyses avec vos collègues ? | | oui | | non | | | je ne sais pas le faire |
|  |  | 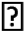 | | 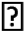 | | | 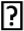 |
| 4. Savez-vous crée un tableau de bord dans le DHIS 2 ? | | Oui | | Non | | | Ne sait pas |
|  |  | 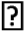 | | 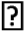 | | | 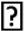 |
| Si oui, pourriez-vous me montrer celui que vous avez crée ? | | | | | | |  |
| 1. **Saisie des fiches de notification/investigation** | | | | | | |  |
| 5. Avez-vous suivi un cas suspect d’une MPE qui a nécessité un prélèvement pendant la phase test ? | | Oui | | Non | | | Ne sait pas |
|  |  | 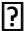 | | 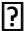 | | | 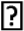 |
| 6. Si oui, Avez-vous suivi le processus pour cette MPE depuis la notification jusqu’au prélèvement labo pour un ou plusieurs cas ? | | Oui | | Non | | | Ne sait pas |
|  |  | 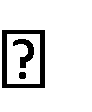 | | 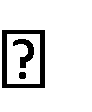 | | | 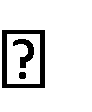 |
| 7. Est-ce que chaque cas notifié dans le SAP a une fiche de notification saisie dans le DHIS 2 ? | | Oui | | Non | | | Ne sait pas |
|  |  | 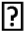 | | 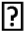 | | | 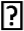 |
| 8. Si oui pour la MPE que vous avez suivi le nombre de fiches manquantes est-il connu ? | | Oui | | Non | | | Ne sait pas |
|  |  | 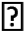 | | 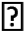 | | | 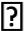 |
| 9. Si oui de quel maladie s’agit-il ?  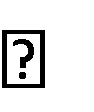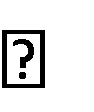Choléra Fièvre jaune Ebola Méningite Rougeole 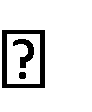 Paralysie Flasque Aigue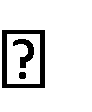 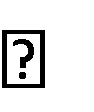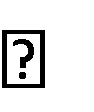 quel circuit les données avaient-elles suivies ?……………………………………………………………………………………………………………  ………………………………………………………………………………………………………………………………………………………………………………….. | | | | | | | |
| 10. Pour les cas notifiés et échantillons envoyés au laboratoire dans les 2 dernières semaines, est-ce que les résultats de labo ont été transmis ? | oui pour tous les cas | | oui, pour la majorité des  cas | | | oui, pour quelques-uns des cas | |
|  | 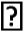 | | 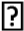 | | | 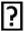 | |
|  | non, aucun des résultats n’a été transmis | | Non je ne sais pas où trouver les résultats | | | je ne sais pas | |
|  | 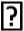 | | 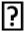 | | | 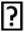 | |
| 11. Les laboratoires saisissent-ils les résultats des tests dans les fiches de notification du DHIS 2 Tracker ? | Oui | | non | | | Ne sait pas | |
|  | 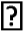 | | 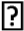 | | | 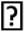 | |
| 12. Comment est-ce que les MCMs/DPS reçoivent  les résultats des tests de laboratoire sur les cas qu’ils ont notifié ? | à travers le DHIS 2 | | Au téléphone | | | par l’ANSS | |
|  | 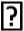 | | 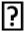 | | | 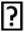 | |
|  | Une lettre | | A travers l’agent de l’OMS | | | autre moyen | |
|  | 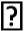 | | 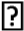 | | | 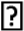 | |
| Au cas où ils reçoivent le résultat à travers une autre organisation, veillez mentionner le nom de cette organisation:…………………………………………………………..…………………………………………………………………………………… | | | | | | | |
| 1. **Validation** | **des données** | |  | | |  | |
| 13. Vérifiez-vous la qualité des données dans le DHIS 2 ? | Oui | | non | | | Ne sait pas le faire | |
|  | 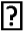 | | 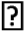 | | | 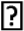 | |
| 14. Avez-vous constatés des erreurs de saisie dans le DHIS 2 ? | Oui | | non | | | Ne sait pas | |
|  | 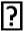 | | 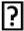 | | | 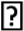 | |
| 15. Avez-vous constaté la violation des règles de validation ? | Oui | | non | | | Ne sait pas | |
|  | 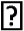 | | 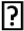 | | | 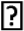 | |
| 16. Avez-vous observé des données aberrantes lors de l’exploitation des données dans le DHIS 2? | Une fois | | Deux fois | | Aucune | | |
|  | 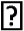 | | 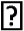 | | 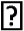 | | |
| 17. Depuis la phase test du DHIS 2 combien de mission de supervision avez-vous effectué pour valider les données remontées ? | Une fois | | Deux fois | | Aucune | | |
|  | 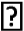 | | 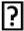 | | 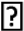 | | |
| 18. Comment validez-vous les données remontées par les DPS ? | Au téléphone | | Lors des CTPS | | Lors des missions de supervisions | | |
|  | 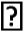 | | 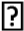 | | 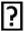 | | |
| 1. **Conditions de fonctionnement du DHIS 2** | | | | | | | |
| 19. L’institution dispose-t-elle d’équipement informatique (ordinateur) fonctionnel? | Oui | | Non | | Ne sait pas | | |
|  | 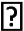 | | 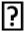 | | 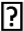 | | |
| 20. dispose-t-elle d’une source d’énergie fonctionnelle? | Oui | | Non | | Ne se sait pas | | |
|  | 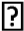 | | 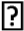 | | 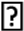 | | |
| 21. dispose-t-elle d’une connexion internet fonctionnelle ? | Oui | | Non | | Ne se sait pas | | |
|  | 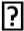 | | 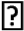 | | 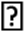 | | |
| 22. Combien de fois depuis 1 juin 2017 avez-vous eu des coupures de courant qui ont empêché l’utilisation de DHIS 2 ? | (1) 10 fois ou plus | | 2) 5 fois ou plus | | 3) 2 fois ou plus | | |
|  | 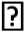 | | 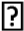 | | 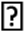 | | |
|  | 4) 1 fois ou plus | | ) je n’ai pas eu de coupure de courante qui a  empêché de  travaille dans le DHIS 2 pendant la période | | je ne sais pas | | |
|  | 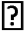 | | 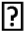 | | 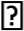 | | |
| 23. Combien de fois depuis le 1^er^ juin 2017 avez-vous eu des coupures d’internet qui ont empêché l’utilisation de DHIS 2 ? | 10 fois ou plus | | 5 fois ou plus | | 2 fois ou plus | | |
|  | 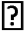 | | 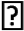 | | 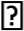 | | |
|  | 1 fois ou plus | | je n’ai pas eu de coupure | | je ne sais pas | | |
|  | 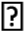 | | 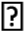 | | 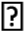 | | |
|  | d’internet qui a empêché de travaille dans le DHIS 2 pendant la période | | Pas de réponse | | Autres (à préciser) | | |
|  | 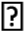 | | 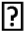 | | 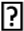 | | |
| 1. **Analyse des avantages et inconvénients du DHIS2** | | | | | | | |
| 24. A votre avis est-ce que l’utilisation du DHIS 2 pour la surveillance épidémiologique apporte des avantages par rapport au système existant? | oui | | non | | Je ne sais pas | | |
|  | 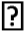 | | 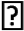 | | 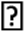 | | |
| 48. Si oui quels sont les avantages que vous avez remarqués? | Facilite l’analyse des données | | Facilite la saisie des données | | Facilite le partage des données | | |
|  | 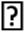 | | 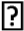 | | 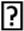 | | |
|  | Permet de recevoir les résultats du labo | | Permet de comparer les données | | Facilite l’élaboration des graphiques | | |
|  | 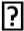 | | 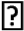 | | 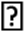 | | |
|  | Facilite la prise de décision | | Offre la possibilité d’avoir des données exhaustives à tous les niveaux | | Offre la possibilité d’avoir des données harmonisées par niveau | | |
|  | 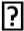 | |  | |  | | |
|  | N’a pas encore fait les comparaisons | | Ne sait pas | | Autres | | |
|  |  | |  | |  | | |
| 49. Si non l’utilisation du DHIS2 est-elle la même ou moins efficace que le système existant | La même | | Moins efficace | | Plus efficace | | |
|  |  | |  | |  | | |
| 50. Si vous pensez que c’est moins efficace quelles sont les raisons ? | Trop difficile à utiliser | | Manque  d’internet | | Manque de courant | | |
|  |  | |  | |  | | |
|  | Il n’y a pas les informations dont j’ai besoins | | Connection lente | | Manque de formation | | |
|  |  | |  | |  | | |
| Autre à préciser | | | | | | | |
| 51. Comment avez-vous participé à la phase pilote du DHIS 2?  (cocher toutes les réponses applicables, plusieurs réponses sont possibles) | j’ai participé aux visites de supervision | | j’ai fait le  suivi /appuyé les utilisateurs à distance | | j’ai regardé les données de SAP /et ou fiches de notification dans le DHIS 2 | | |
|  |  | |  | |  | | |
|  | j’ai assisté aux réunions de la phase pilote | | j’ai assisté aux formations des utilisateurs | | Autre (à spécifier) | | |
|  |  | |  | |  | | |
| 52. Si non pour quelles raisons ? | Manque de temps | | Pas très concerné | | Pas d’avis | | |
|  |  | |  | |  | | |
| 53. La méthodologie de la formation répond-elle aux besoins des utilisateurs ? | Oui | | Non | | Pas de réponse | | |
|  |  | |  | |  | | |
| 54. Quels changements doivent être faits sur le plan de la formation ? | Le temps de formation est court | | Réduire le nombre de personnes par séance | | Aucun changement | | |
|  |  | |  | |  | | |
| 55. Quels changements doivent être faits dans le contenu de la formation | Faire beaucoup plus d’analyses | | Donner beaucoup d’exercices pratiques | | Aucun changement | | |
|  |  | |  | |  | | |

Commentaire général:

........................................................................................................................................................................

........................................................................................................................................................................

.......................................................................................................................................................................

........................................................................................................................................................................ ........................................................................................................................................................................

MERCI BEAUCOUP POUR VOTRE DISPONIBILITE ET LA FRANCHISE DE VOS REPONS

**2. Questionnaire for Regional and District Level Health Personnel**

**QUESTIONNAIRE D’EVALUATION DE LA PHASE PILOTE DU MODULE SURV EPI DU DHIS 2**

Numéro du questionnaire: [__|___|__]___]

Informations personnelles

Nº [__|___|__]

Date de l’enquête: [__|__] /[__|__]/[__|__]

Mon nom est…………………………………………au nom du Ministère de la Santé. Nous sommes en train de collecter des informations pour évaluer les activités du DHIS 2. Nous vous prions de répondre aux questions afin de nous aider à mieux comprendre les acquis et les besoins d’améliorations à apporter avant la mise à l’échelle du module dans le reste du pays.

Vos réponses seront tenues strictement confidentielles. Votre nom ne sera pas inscrit sur le questionnaire et aucun lien ne pourra être fait entre ce que vous me dites et votre nom. Vous n’êtes pas obligé(e) de répondre à une question et vous pouvez arrêter l’entretien à tout moment Votre participation est volontaire et cet entretien ne durera pas plus de 60 minutes.

Je certifie que l’enquêté(e) a été informé(e) de la nature, du but de l’évaluation et qu’il (elle) a donné son consentement pour participer dans cette évaluation.

Signature de l’enquêtée : ___________________________________________

Signature de l’enquêteur : ___________________________________________

Date de la signature de la fiche d’enquête par l’enquêteur : [__|__] /[__|__]/[__|__]

*A TOUS LES ENQUETEURS : VOUS VEILEREZ A ENCERCLER, COCHER ET/OU INSCRIRE LA REPONSE DES REPONDANTS A LA PLACE PREVUE A CET EFFET.*

**INFORMATIONS GENERALES SUR LA PERSONNE INTERVIEWEE AU NIVEAU REGIONAL/PREFECTORAL**

1. Région de : .................................................................................................................... 2. Préfecture de :............................................................................................................... 3. Structure: ......................................................................................................................

1. Poste occupé dans la structure:....................................................................................
2. Personne formée : Oui non
3. Date de la formation : …………………………………………………………………………………………
4. Nombre de structures en charge dans la surveillance: ............................................
5. Nombre de centres de santé couverts par le District sanitaire : ................................
6. Nombre de structures privées évoluant dans le District Sanitaire : ...........................
7. Date de l’entretien:......................................................................................................

**Analyse des aptitudes individuelles acquises dans l’exploitation du DHIS 2**

**(Veillez demander à la personne interviewée de faire la pratique, observez et noter au fur et à mesure de l’application du questionnaire) NB 1 = oui et 0=non**

| ***Capacités individuelles de la personne enquêtées dans la saisie et l’analyse des données du DHIS 2*** | Réponse | Observations |
| --- | --- | --- |
| 1- Avez-vous saisie ou vérifié les données dans le DHIS 2 ? Si oui, pourriez-vous me montrer un exemple ? | …………/ 1 |  |
| 2- Avez-vous saisie ou vérifié les données SAP hebdomadaires dans le DHIS 2 ? Si oui, pourriez-vous me montrer un exemple ? | …………/ 1 |  |
| 3- Avez-vous saisie ou vérifié des fiches de décès maternels dans le DHIS 2 ? Si oui, pourriez-vous me montrer un exemple ? | …………/ 1 |  |
| 4- Avez-vous saisie ou vérifié des fiches individuelles de notification/investigation dans le DHIS 2 ? Si oui, pourriez-vous me montrer un exemple ? | …………/ 1 |  |
| 5- connaissez-vous la différence entre le bouton « terminer » et le bouton « incomplet » | …………/ 1 |  |
| 6- connaissez-vous comment changer de navigateur en cas de problème? | …………/ 1 |  |
| 7- Pouvez-vous nous expliquer comment préparer un rapport de complétude | …………/ 1 |  |
| 8- Au cas où vos crédits de connexion sont finis, savez-vous comment activer les crédits et vérifier la consommation ? | …………/ 1 |  |
| 9- Savez-vous comment visualiser le tableau de bord (faites une démonstration SVP) ? | …………/ 1 |  |
| 10- Pouvez-vous faire une démonstration de l’utilisation des modules d’analyse SAP | …………/ 1 |  |
| 11- savez-vous sauvegarder dans le favori d’un tableau de bord que vous avez créez ? | …………/ 1 |  |
| 12- Savez-vous Ecrire un message à travers DHIS2 et envoyer aux autres utilisateurs ? | …………/ 1 |  |
| Total (sur 12) | …………/ 12 |  |

| **Questions et filtres** | | | | **Réponses** | | | | | | | |
| --- | --- | --- | --- | --- | --- | --- | --- | --- | --- | --- | --- |
| 1. **Analyse des données** | | | | | | | | | | | |
| 1. Avez-vous regardé ou analysé les données de surveillance épidémiologique dans le DHIS 2 ?   (si non, ou pas de réponse, aller a la question 6) | | | | Oui | Non | | | | | Pas de réponse | |
|  |  |  |  |  |  | | | | |  | |
| 2. Si Oui, quels outils dans le DHIS 2 avez-vous utilise pour les analyse de données ? (Cochez toutes les réponses mentionnées par le répondant)  **ne lisez pas les réponses possibles** | | | | tableau croisé  dynamique | Système d’Information Géographique (SIG) | | | | | visualiser de données | |
|  |  |  |  |  |  | | | | |  | |
|  |  |  |  | rapports d’évènements | rapports standards | | | | | Visualiser d’évènement | |
|  |  |  |  |  |  | | | | |  | |
| 4. Si vous avez fait une analyse de données dans le DHIS 2, avez-vous partage le résultat de vos analyses, (le tableau croisé dynamique, la carte ou le tableau de bord etc.) avec les autres collègues ? | | | | oui | non | | | | | Pas de réponse | |
|  |  |  |  |  |  | | | | |  | |
| 5. Avez-vous crée un tableau de bord dans le DHIS 2 ? | | | | Oui | Non | | | | | Pas de réponse | |
|  |  |  |  |  |  | | | | |  | |
| Si oui, pourriez-vous me le montrer | | | | |  | | | | |  | |
| 1. **Saisie des fiches de notification/investigation** | | | | |  | | | | |  | |
| 6. Avez-vous eu au moins un cas suspect avec une fiche de notification qui a demandé un prélèvement pendant la phase pilote ? | | | | Oui | Non | | | | | pas de réponse | |
|  |  |  |  |  |  | | | | |  | |
| 7. chaque cas notifié a-t-elle une fiche de notification saisie dans le DHIS 2 ? | | | | Oui | Non | | | | | pas de réponse | |
|  |  |  |  |  |  | | | | |  | |
| 8. Si oui de quel (s) maladie(s) s’agit-il ?  Choléra Fièvre jaune Ebola Méningite Rougeole Paralysie Flasque Aigue  quel circuit les formulaire (s) ont ils suivies ?………………………………………………………………………………………………………………………………………………………………………… | | | | | | | | | | | |
| 9. Pour les cas notifiés et échantillons envoyés au labo au cours des 2 dernières semaines, est-ce que vous avez reçus les résultats de labo ? | oui pour tous les cas | | | | oui, pour la majorité des  cas | | | | | oui, pour quelques-uns des cas | |
|  |  | | | |  | | | | |  | |
|  | non, je n’ai reçu aucun des résultats | | | | Non je ne sais pas où trouver les résultats | | | | | pas de réponse | |
|  |  | | | |  | | | | |  | |
| Autres à préciser : | | | | | | | | | | | |
| 10. Les laboratoires saisissent-ils les résultats de tests dans les fiches de notification du DHIS 2 Tracker ? | Oui | | | | non | | | | | pas de réponse | |
|  |  | | | |  | | | | |  | |
| 11. Comment est-ce que vous recevez les résultats laboratoires des prélèvements effectués? | à travers le DHIS 2 | | | | Au téléphone | | | | | par le niveau central | |
|  |  | | | |  | | | | |  | |
|  | à travers une lettre | | | | A travers l’agent de l’OMS | | | | | par téléphone/email/SMS d’une autre organisation | |
|  |  | | | |  | | | | |  | |
|  | Par téléphone email/SMS du labo | | | | par téléphone/email/SMS du niveau central | | | | | j’ai consulté le résultat dans le  DHIS 2 | |
|  |  | | | |  | | | | |  | |
|  | je n’ai reçu aucun résultat labo pendant la phase test du  DHIS 2 | | | | autre moyen | | | | | Pas de réponse | |
|  |  | | | |  | | | | |  | |
| Au cas où vous avez reçu le résultat à travers une autre organisation, veillez mentionner le nom de cette organisation | | | | | | | | | | | |
| :…………………………………………………………..……………………………………………………………………………… | | | | | | | | | | | |
| 1. **Validation des données** | | | | | | | | | | | |
| 12. Depuis le 1^er^ juin combien de missions de supervision avez-vous effectué pour valider les données SAP et notification remontées par les  CS (point focal DHIS2/MCM) | Une fois | | | | Deux fois | | | | | Aucune | |
|  |  | | | |  | | | | |  | |
|  | Plus de deux fois | | | | Plus de trois fois | | | | | Pas de reponse | |
|  |  | | | |  | | | | |  | |
| 13. Comment vous assurez-vous de la cohérence des données (SAP, notification) remontées par les CS ? | Au téléphone | | | | Lors des CTPS | | | | | Lors des missions de supervisions | |
|  |  | | | |  | | | | |  | |
|  | Lors des réunions hebdomadaires | | | | Lors des réunions mensuelles | | | | | Autre a preciser | |
|  |  | | | |  | | | | |  | |
|  | | | | | | | | | | | |
|  | | | | | | | | | | | |
| 1. **Conditions de fonctionnement du DHISS 2** | | | | | | | | | | | |
| 14. La DPS dispose-t-elle d’équipement informatique (ordinateur) fonctionnel? | | Oui | | | | Non | | | Pas de réponse | |  |
|  |  |  | | | |  | | |  | |  |
| 15. La DPS dispose-t-elle d’une source d’énergie fonctionnelle? | | Oui | | | | Non | | | Pas de réponse | |  |
|  |  |  | | | |  | | |  | |  |
| 16. La DPS dispose-t-elle d’une connexion internet fonctionnelle ? | | Oui | | | | Non | | | Pas de réponse | |  |
|  |  |  | | | |  | | |  | |  |
| 18. Combien de fois depuis le 1^er^ juin 2017 avez-vous enregistré une interruption du service par manque d’énergie qui a empêché l’utilisation de DHIS 2 ? | | 10 fois ou plus | | | | 5 fois ou plus | | | 2 fois ou plus | |  |
|  |  |  | | | |  | | |  | |  |
|  |  | 1 fois ou plus | | | | je n’ai pas eu de coupure de courante qui a  empêché de  travaille dans le DHIS 2 pendant la période | | | Pas de réponse | |  |
|  |  |  | | | |  | | |  | |  |
| 19. Combien de fois depuis le 1 juin 2017 avez-vous eu des coupures d’internet qui ont empêché l’utilisation de DHIS 2 ? | | 1) 10 fois ou plus | | | | (; 2) 5 fois ou plus | | | 3) 2 fois ou plus | |  |
|  |  |  | | | |  | | |  | |  |
|  |  | 4) 1 fois ou plus | | | | 5) je n’ai pas eu de coupure d’internet qui  a empêché de travaille dans le DHIS 2 pendant la période | | | 6) je ne sais pas | |  |
|  |  |  | | | |  | | |  | |  |
| 1. **Analyse des avantages et inconvénients du DHIS2** | | | | | | | | | | |  |
| 20. A votre avis est-ce que l’utilisation du DHIS 2 pour la surveillance épidémiologique apporte des avantages par rapport au système existant? | | oui | | | | non | | | Pas de réponse | |  |
|  |  |  | | | |  | | |  | |  |
| 21. Si oui quels sont les avantages que vous avez remarqués? | | | Facilite l’analyse des données | | | | Facilite la saisie des données | Facilite le partage des données | | |  |
|  |  |  |  | | | |  |  | | |  |
|  |  |  | Permet de recevoir les résultats du laboratoire | | | | Permet de comparer les données | Autres (à préciser) | | |  |
|  |  |  |  | | | |  |  | | |  |
| 22. Si non l’utilisation du DHIS2 est-elle la même ou moins efficace que le système existant | | | La même | | | | Moins efficace | Plus efficace | | |  |
|  |  |  |  | | | |  |  | | |  |
| 23. Si vous pensez que c’est moins efficace quelles sont les raisons ? | | | Trop difficile à utiliser | | | | Manque  d’internet | Manque de courant | | |  |
|  |  |  |  | | | |  |  | | |  |
|  |  |  | Il n’y a pas les informations dont j’ai besoins | | | | Connection lente | Manque de formation | | |  |
|  |  |  |  | | | |  |  | | |  |
| Autre à préciser | | | | | | | | | | |  |
| 24. Avez-vous participe a la phase pilote du DHIS 2 ? Oui Non | | | | | | | | | | |  |
| 25. Comment avez-vous participe à la phase pilote du DHIS 2 ? (crocher tous les réponses applicables (plusieurs réponses sont possibles) | | | 1) j’ai participé aux visites de supervision | | | | 2) j’ai fait le  suivi /appuyé les utilisateurs à distance | 3) j’ai regardé les données de SAP /et ou fiches de notification dans le DHIS 2 | | |  |
|  |  |  |  | | | |  |  | | |  |
|  |  |  | 4) j’ai assisté aux réunions sur la phase pilote | | | | 5) j’ai assisté aux formations des utilisateurs | 6) autre (à spécifier) | | |  |
|  |  |  |  | | | |  |  | | |  |
| 26. Si non pour quelles raisons ? | | | Manque de temps | | | | Pas très concerné | Pas d’avis | | |  |
|  |  |  |  | | | |  |  | | |  |
|  |  |  | Manque de ressources | | | | Pas informé pour les visites de supervisions | Autres a preciser | | |  |
|  |  |  |  | | | |  |  | | |  |
| 27. Est-ce que la méthodologie de la formation répond aux besoins des utilisateurs | | | Oui | | | | Non | Pas d’avis | | |  |
|  |  |  |  | | | |  |  | | |  |
| 28. Quels changements doivent être faits sur le plan/approche de formation ? | | | Le temps de formation est court | | | | Réduire le nombre de personnes par séance | Aucun changement | | |  |
|  |  |  |  | | | |  |  | | |  |
| 29. Quels changements doivent être faits dans le contenu de la formation | | | Faire beaucoup plus d’analyses | | | | Beaucoup d’exercices pratiques | Aucun changement | | |  |
|  |  |  |  | | | |  |  | | |  |
| 30. es suivis réalisés ont–ils été efficace ? | | | Oui | | | | Non | Pas de reponse | | |  |
|  |  |  |  | | | |  |  | | |  |

Commentaire général

...........................................................................................................................................................................................

............................................................................................................................................................................................ ............................................................................................................................................................................................

MERCI BEAUCOUP POUR VOTRE DISPONIBILITE ET LA FRANCHISE DE VOS REPONSES

1. Membre de l’équipe d’évaluation :

| Nom et Prénom (s) | Fonction | Contact | Emargement |
| --- | --- | --- | --- |
|  |  |  |  |
|  |  |  |  |
|  |  |  |  |
